# Discovering Disease Associations by Integrating Electronic Clinical Data and Medical Literature

**DOI:** 10.1371/journal.pone.0021132

**Published:** 2011-06-23

**Authors:** Antony B. Holmes, Alexander Hawson, Feng Liu, Carol Friedman, Hossein Khiabanian, Raul Rabadan

**Affiliations:** 1 Department of Biomedical Informatics, Columbia University College of Physicians and Surgeons, New York, New York, United States of America; 2 Center for Computational Biology and Bioinformatics, Columbia University College of Physicians and Surgeons, New York, New York, United States of America; 3 Department of Medicine, Columbia University College of Physicians and Surgeons, New York, New York, United States of America; University of Chicago, United States of America

## Abstract

Electronic health record (EHR) systems offer an exceptional opportunity for studying many diseases and their associated medical conditions within a population. The increasing number of clinical record entries that have become available electronically provides access to rich, large sets of patients' longitudinal medical information. By integrating and comparing relations found in the EHRs with those already reported in the literature, we are able to verify existing and to identify rare or novel associations. Of particular interest is the identification of rare disease co-morbidities, where the small numbers of diagnosed patients make robust statistical analysis difficult. Here, we introduce ADAMS, an Application for Discovering Disease Associations using Multiple Sources, which contains various statistical and language processing operations. We apply ADAMS to the New York-Presbyterian Hospital's EHR to combine the information from the relational diagnosis tables and textual discharge summaries with those from PubMed and Wikipedia in order to investigate the co-morbidities of the rare diseases Kaposi sarcoma, toxoplasmosis, and Kawasaki disease. In addition to finding well-known characteristics of diseases, ADAMS can identify rare or previously unreported associations. In particular, we report a statistically significant association between Kawasaki disease and diagnosis of autistic disorder.

## Introduction

The latter half of the twentieth century and to a greater extent the beginning of the twenty-first century saw a prolific and rapid expansion in the transition of clinical record systems from paper to electronic. Health care systems stand to benefit signficantly from the switch to electronic health record (EHR) systems as more clinical data become more easily captured and accessed. The complex nature of health care and of how patient information is reported makes EHR systems extremely challenging to implement. We are only now beginning to see their power and to learn how they can be effectively mined for information. Governments are pushing for the adoption of EHR systems because of their burgeoning importance in effective care and treatment. In the United States, for example, the 2009 American Recovery and Reinvestment Act has allocated $19 billion to research into health information systems [1], and in the United Kingdom, a large project to modernise the National Health Service and to move all patient records to a centralized database is currently underway [2]–[4]. It is important to understand how the large amounts of data being archived in these systems can be correctly and effectively interpreted.

EHRs not only facilitate improvements in quality of care [1], they also facilitate clinical research and epidemiological studies, particularly as they increase the availability of patients' medical information [5], [6]. The Rare Disease Act of 2002 defines a rare disease as any disease or condition that affects fewer than two hundred thousand persons in the United States [7]. Therefore, by definition, there are a minimal number of people suffering from any particular rare condition, making rigorous statistical analyses of these patient cohorts complex. EHRs offer some of the most enriched data sets available for studying rare diseases in detail, in particular for studying associated risks to determine if there are factors that may exacerbate conditions.

**Figure 1:**
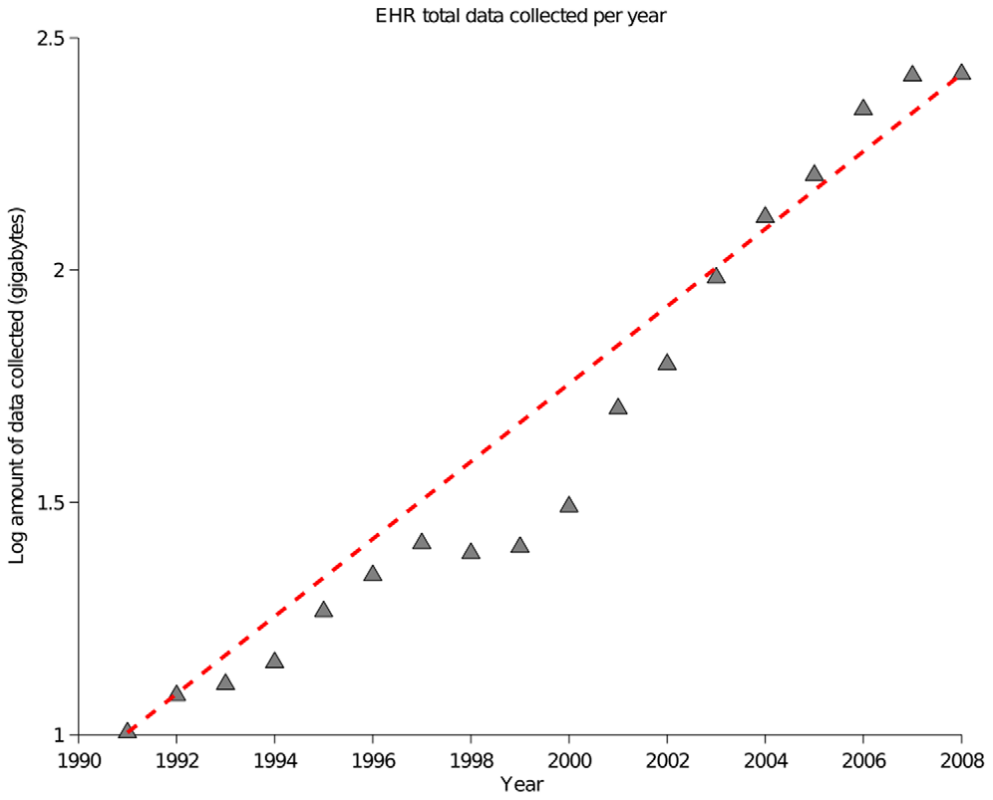
The amount of data entered into the NYPH EHR database each year has been increasing at an exponential rate since 1990 with data entry doubling every 8 years.

The New York-Presbyterian Hospital (NYPH) has been using an electronic health record system for the past twenty years. Each patient gets a descriptive, longitudinal record describing what happened during each patient encounter. The record details attending consultations, case histories, diagnoses, lab test results, medications, and procedures. Therefore the EHR at NYPH has become the de facto method for storing information about patients and an important means of carrying out analyses of patient data. The amount of data entered into the NYPH EHR has been increasing at an exponential rate, with data entry doubling every eight years (Fig. 1), making it a large and comprehensive EHR system.

Finding information on rare diseases in the NYPH EHR is a data mining problem. Processing several hundred million records requires an automated extraction and classification process. The NYPH EHR system can be loosely divided into two parts: one that archives physician notes in a textual format, and one that describes patients' states using code descriptors in the form of proprietary internal codes or codes from the International Classification of Diseases, Ninth Revision, Clinical Modification (ICD-9-CM). Textual notes provide the greatest amount of information about a patient; however, they contain natural language, which can be ambiguous and context-based with relatively complex grammar, thus making accurate information extraction hard. ICD-9-CM codes, on the other hand, are easy to process, but they are not necessarily comprehensively descriptive. Missing and incomplete data also make discerning signal more difficult, since EHRs may not be fully up to date, depending on the frequency of patient visits.

Textual sources can be data mined to understand whether local EHR findings have been observed elsewhere. Journal articles can complement EHR data for studying disease associations by providing an aggregate of published information from around the world. Data mining journal articles has become a feasible method for determining relationships between concepts [8]. The National Center for Biotechnology Information (NCBI) maintains PubMed, an extensive Internet-based journal archive of biological papers [9]. Data mining and natural language processing (NLP) have previously been used to analyze journal articles [10]–[13]. Web sites such as Wikipedia provide another source of information on diseases and have recently started to be data mined [14], [15]. Although considered less reliable than journal sources, web sites contain data on many subjects and serve as a proxy to summarize multiple sources.

Previous studies have used EHR systems to research diseases; however, they primarily use the EHR as the sole data source (for example see [16]–[18]). In this paper, we present ADAMS, an Application for the Discovery of Disease Associations using Multiple Sources. It combines rigorous multiple hypothesis testing and false discovery rate analysis of relational diagnosis tables and NLP processed textual discharge summaries, in addition to the information retrieved from PubMed and Wikipedia. We apply ADAMS to the NYPH EHR to investigate the co-morbidities associated with Kaposi sarcoma, toxoplasmosis, and Kawasaki disease.

Kaposi sarcoma is a tumor caused by human herpesvirus 8, also known as Kaposi sarcoma-associated herpesvirus [19]. The condition came to prominence with the AIDS epidemic of the 1980's as it was seen to be a frequent and aggressive disease in AIDS patients. It also has been closely associated with immunosuppressed patients [20], [21]. We chose to apply our methodology to Kaposi sarcoma because of the high risk and important role it plays in patients with HIV/AIDS.

Toxoplasmosis also affects immunocompromised individuals, either through HIV infection or immunosuppressive therapy. It is caused by the obligate intracellular parasitic protozoon *toxoplasma gondii*. It was selected to study whether the signs and symptoms from congenital infection and immunocompromise could be identified. Furthermore, comparing Kaposi sarcoma with toxoplasmosis could allow for the study of ICD-9-CM codes specific to each as opportunistic infections [22].

We also selected a rare disease for its peak incidence in a narrow age group. Kawasaki disease, also known as mucocutaneous lymph node syndrome, is an acute vasulitis that most commonly occurs between one and four years of age. It is generally benign and self-limited, although it is associated with coronary artery aneurysms and an overall case-fatality rate of 0.5–2.8% [23]–[25]. This disease was selected because of the predominant distribution in children and to evaluate ADAMS's ability to identify predisposing risk factors and sequelae of the disease.

To account for background noise in the data, control groups were required, for which we chose cohorts of post-traumatic stress disorder (PTSD) patients and influenza patients. PTSD is a psychiatric condition that can affect people who experience traumatic events. PTSD is a fitting control disease because it is regarded as the “common cold” of psychiatric disorders [26]. Exposure is common, with two-thirds of the population exposed to a traumatic event at least once throughout their lives. Most people who experience traumatic events will not develop PTSD, as the lifetime prevalence of PTSD in the general population is around 7%. Half of the people who develop PTSD recover within a year without professional help, but 15% will not recover despite receiving treatment [27].

Influenza patients are suitable as a control cohort since influenza is a broadly contracted infectious disease. While influenza virus can infect individuals without causing acute symptomatic disease, some people do suffer sufficiently acute symptoms to visit the hospital. These people who come to the hospital would be sicker than those who can stay home; the NYPH EHR would have a bias toward sicker influenza patients. Since influenza is a control, this bias would make statistically significant case associations less likely among the ICD-9-CM codes that occur more often among sicker influenza patients.

It is a difficult problem to define a representative background control set of patients against which to compare a case disease. Ideally the background would be as comprehensive as the case histories of every person in the world; however, we are limited by the information present in the EHR and its biases. Using two control cohorts with different etiologies should expose most data biases within each control by comparing each case cohort to both. Although less than the ideal, being aware of each control group's biases will allow for broader and more nuanced analyses of the case diseases.

## Methods

ADAMS is an application that can compare case and control disease cohorts within an EHR and then generate statistical analyses that can be compared to external data sources. Based on each case-control pairing, ADAMS can generate a list of all ICD-9-CM codes within the case cohort along with their respective p-values and false discovery rates (FDR). At any given FDR threshold, ADAMS can generate a network diagram of statistically significant ICD-9-CM codes that can be tied to other data sources. The data sources used for this paper were:

1. The NYPH EHR ICD-9-CM coded diagnoses;
2. The NYPH EHR natural language processed discharge summaries;
3. Article abstracts mentioning the rare diseases from the National Center for Biotechnology Information (NCBI) PubMed repository;
4. Wikipedia articles on the rare diseases.

### Diagnosis ICD-9-CM tables

The NYPH IRB protocol for this project was marked as Non Human Subject Research and thus was exempt from the requirement of formal approval by the IRB. The NYPH EHR was de-identified in accordance with HIPAA regulations, and all data that could uniquely identify patients were removed before the study was commenced. This limited data set comprises multiple tables containing diagnoses, procedures, lab results, prescription orders, and demographic information encoded using ICD-9-CM codes [28] and proprietary medical codes. ICD-9-CM codes form a hierarchy to describe conditions in increasingly finer granularity. The codes are limited in expression and do not describe all conditions. They are not always accurate indicators of medical conditions [29], [30]; but in the case of the diseases discussed in this paper, which generally have specific, well-defined names, we assume that the ICD-9-CM codes are accurately assigned to patients because the code descriptions clearly contain the disease names (Table 1). Incomplete data inherent within the EHR mean that the number of patients from this data set provides the lower bound for the actual number of patients at NYPH with each disease.

**Table 1:** ICD-9 codes used to retrieve the sets of patients of the three rare diseases and the two control groups from NYPH EHR (2004–2009).

Rare disease	ICD-9 codes	Number of patients
Kaposi sarcoma	176, 176.0, 176.1, 176.2, 176.3, 176.4, 176.5, 176.8, 176.9	221
Toxoplasmosis	130, 130.0, 130.1, 130.2, 130.3, 130.4, 130.5, 130.7, 130.8, 130.9	138
Kawasaki disease	446.1	213
Post-traumatic stress disorder (control)	309.81	1281
Influenza (control)	487, 487.0, 487.1, 487.8	2582

### Discharge summaries

**Figure 2:**
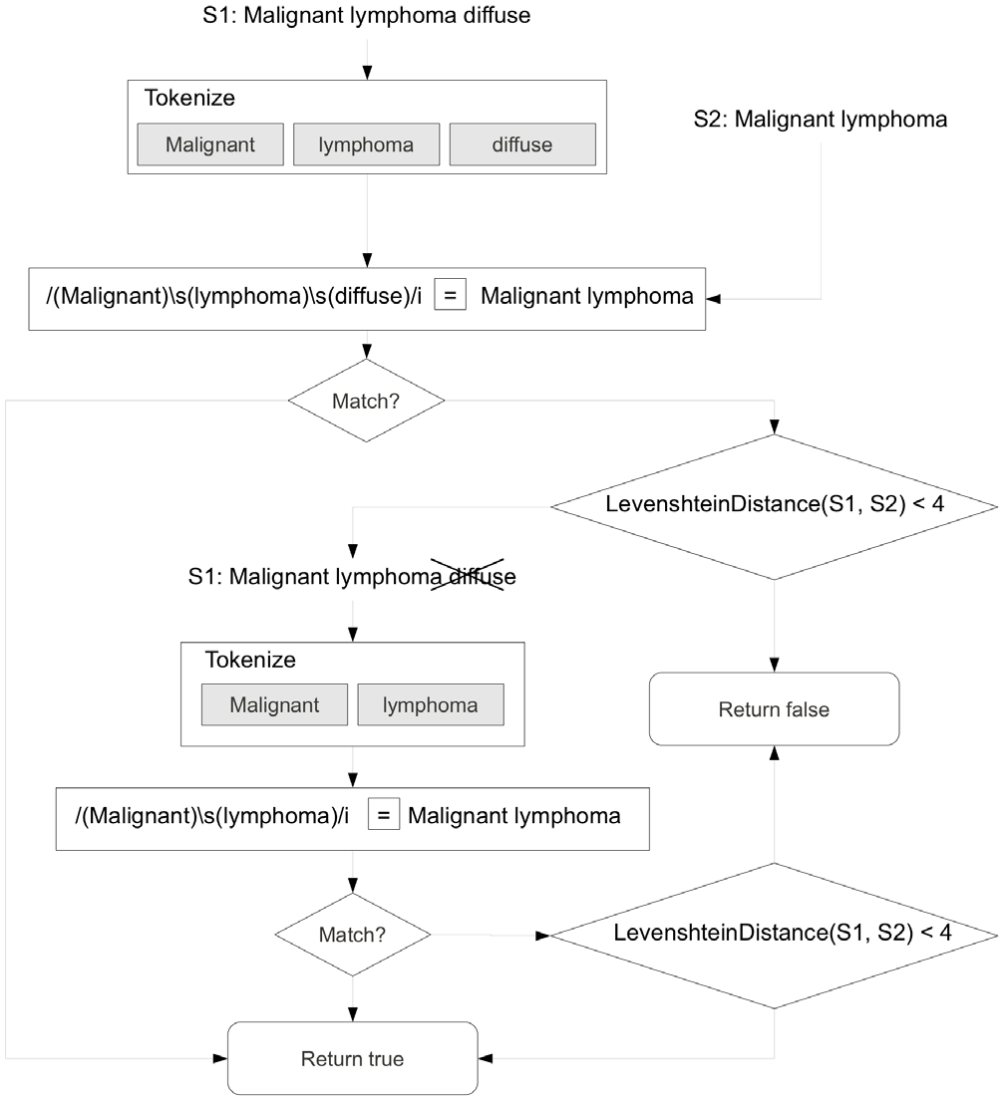
Outline of how search terms are mapped against ICD-9 code descriptions, using malignant lymphoma as an example. Firstly the search term (S1) is broken down into words which are matched against the target phrase (S2) using regular expressions. Starting with every word in S1, S1 and S2 are compared and if there is no match, words are repeatedly removed from the match expression until only one word remains. If no match is found, a Levenshtein distance function is used to compare the terms for equality and if it scores lower that a threshold 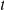 the terms are considered as matching.

In addition to the diagnosis tables, which primarily record out-patient visits, the limited NYPH EHR also includes de-identified in-patient discharge summaries, which were parsed for disease associations. Discharge summaries are textual reports written by physicians. These were processed using Medical Language Extraction and Encoding System (MedLEE), an NLP system [31]. The NLP reports contain disease terms found within the discharge summaries that were coded using the National Library of Medicine (NLM) Unified Medical Language System (UMLS) [32]. The discharge summary data were made available at an aggregated level. A search was done to identify the discharge summaries that contained the UMLS codes of the diseases of interest. After the reports were identified, all of the unique UMLS codes across the reports were listed. The NIH NLM mappings between ICD-9-CM and UMLS codes were used, but the mappings were not comprehensive. Many UMLS codes are unmapped to ICD-9-CM codes, and many ICD-9-CM codes are unmapped to UMLS codes [33]. In order to capture the unmapped codes, the UMLS codes were mapped to ICD-9-CM codes using the procedure outlined in Fig. 2 so that a consistent description language was used for medical terms. To be as comprehensive as possible, we used the NYPH discharge summaries recorded between 1991 and 2009. Because the discharge summary data were at an aggregated level, it was not possible to combine ICD-9-CM data and discharge summary data at the patient level.

### PubMed articles

The National Center for Biotechnology Information (NCBI) PubMed resource is a centralized Internet repository of biological and medical journal articles [9]. Data mining the site involved collating all articles whose title or abstract mentioned our rare diseases of interest and then searching against a list of diseases (compiled from ICD-9-CM codes) to find co-occurrences of words. Abstracts were chosen as the primary method of determining associations because of time constraints precluding comprehensive natural language processing of the full texts of journal articles. MedLEE was used to extract disease terms from the abstracts. The results of NLP were in the same aggregated output format as for the discharge summary results. UMLS terms were matched to ICD-9-CM codes with the same method used for discharge summaries.

### Wikipedia articles

Wikipedia was used as a proof of concept that can be mined for rare disease information. Pages of case diseases were downloaded and mined automatically using the Wikipedia application programmers interface (API) [34]. We ensured that only the latest revision of each page was downloaded and analyzed for occurrences of ICD-9-CM code descriptions. For a Wikipedia page to be tagged as having a given ICD-9-CM code, an exact match of that code's full description would have to be found in the page's text. Wikipedia was chosen as a source because of its convenient API and its explicit lack of intellectual property restrictions.

### ADAMS methodology

ADAMS functions as a pipeline of discrete, automated stages where each stage enriches data generated from previous stages. The major steps involved in determining disease assocations using our algorithm can be summarized as follows:

1. Identify diseases of interest for both case and control cohorts.
2. Identify patients with diseases of interest.
3. Load the ICD-9-CM code history of patients with diseases of interest into each disease cohort.
4. Run ADAMS to establish statistically significant associations.
5. Search for disease association data in other data sources such as PubMed and NLP discharge summaries.
6. Draw network diagram combining ICD-9-CM data with data from other sources.

Patients with the diseases of interest were identified using ICD-9-CM codes. Any patient whose medical history included any ICD-9-CM codes that denoted a disease of interest within calendar years 2004–2009 were included in the respective disease cohorts. Using ICD-9-CM codes allowed for broader selection of patients since both inpatient and outpatient records in the NYPH EHR have ICD-9-CM codes.

Once a patient was identified as having a disease of interest, all of that patients ICD-9-CM code data from 2004–2009 were loaded into the respective cohort table. These ICD-9-CM lists served as the basis for finding statistically significant positive associations.

On the ADAMS interface (Fig. S3), the case and control cohorts are selected. ADAMS is also able to limit time intervals for data, but for the purposes of this paper no time limits were specified; the entire time period of 2004–2009 was under evaluation.

Within ADAMS, the hypergeometric distribution was used to calculate the p-value of each ICD-9-CM code in the case cohort versus the control cohort. The cumulative distribution function (cdf) was calculated based on the number of patients with a given code and the total number of patients in each cohort. If a code existed in the case cohort but not the control cohort, the control cohort was assigned a code patient count of zero. The p-value for each code (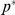) was calculated as (1 - cdf). Because the hypergeometric cdf is asymmetrical, this test identifies positive associations that are statistically significant; negative associations are not identified.

To test for multiple hypotheses, we followed the bootstrapping method described in Khiabanian *et al.* [35]. Bootstrapping involved creating randomized case and control sets based on the observed case and control cohorts. For each case and control cohorts pairing, the case and control patients were placed into a consolidated pool of patients. A patient was selected at random with replacement from the pool. That patient then was assigned to the bootstrap case group. This process was repeated until the bootstrap case group reached the same number of patients as the observed case group. The same process of random selection was done for the bootstrap control group until it reached the same number of patients as the observed control group.

Similar to the previous step, within each bootstrap data set, the hypergeometric value for each ICD-9-CM code was calculated. The number of p-values less than each 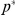 was counted and then divided by the number of bootstraps. This quotient represented the expected count of p-values less than each 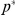. The expected count was divided by the observed count for each 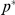. The quotient of expected count divided by observed count defined the false discovery rate (FDR) per 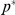. The FDR cutoff was set at less than 0.05. The desired number of bootstraps is entered, which for this paper was one hundred thousand. Clicking on the “Search” button in ADAMS begins the process of statistical analysis.

### Network diagrams

The statistical analysis described thus far applies only to the ICD-9-CM coded data. The other three data sources (discharge summaries, PubMed, and Wikipedia) act as references for comparing patterns observed in the EHR with documented findings in the literature. ADAMS combines the four data sources to create a visual network diagram that draws links between common associations among them. The diagram is ICD-9-CM centric; only the intersections between ICD-9-CM-derived associations and associations from each of the other data sources were included. Thus any associations reported in the other sources that were absent in the ICD-9-CM associations were not shown.

1. Each graph begins with the ICD-9-CM coded set of diseases, 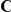, that are associated with the disease of interest.
2. First a node for the disease of interest, 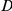, is added to the graph.
3. For each co-occurrence 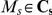, where 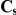 is the set of the statistically significant co-occurrences with 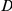 and 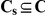, create a graph node for 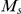 along with an edge connecting 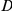 to 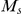.
4. Each of the three ancillary data sources are then queried separately to find the set 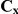 of associated co-occurrences with 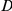.
5. For each co-occurrence 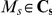 and 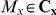, the terms are compared and if a match is found between 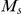 and 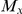, 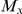 is added as a graph node and an edge between 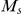 and 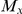 is created.
6. If the nodes and edge do not already exist, a node 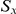 representing the ancillary data source that contains 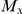 and a connecting edge from 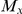 to 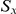 are added.

Despite efforts such as the UMLS to unify ontologies, mapping between different medical vocabularies is non-trivial. Each of the four data sources has its own terminology for particular diseases, so it is not always possible to create perfect mappings between terms. ADAMS uses a fuzzy matching procedure to compare terms in different data sources to reduce the number of unmatched terms (Fig. 2). If an exact match cannot be made, partial terms are made by repeatedly removing individual words from a term and converting plural nouns into their singular form. These partial terms are then matched to each other. Finally, if this fails to yield matches, a Levenshtein distance function [36], [37] is used to calculate the number of insertions, deletions, and changes between the terms; and if this is below a threshold 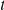, the terms are classed as matching.

The number of records in each data source is given in Table 2.

**Table 2:** Data source sizes.

Data Source	Records
EHR ICD-9-CM patients	768903
NLP records	406158
PubMed Kaposi sarcoma	1025
PubMed Kawasaki disease	598
PubMed Toxoplasmosis	960
Wikipedia	One page per search term, if one exists.

### Adjusting granularity of network diagrams

Although network diagrams contain only statistically significant associations, the number of nodes can still be prohibitively large. To address this issue, custom super nodes can be defined, which subsume existing nodes to adjust the granularity of the network. For example, in the case of Kaposi sarcoma, multiple types of malignant neoplasms can be collapsed into a single “malignant neoplasms” super node (Fig. S1). Super nodes are defined by keywords, against which nodes are matched. As the network is built, each node in a link is compared to the database of super nodes and terms. If a super node match is found, edges are redirected to the super node. Links between super nodes can be suppressed if desired, since this feature can produce misleading links between super nodes when in reality the link occurs because of a node within the category and not the category itself.

## Results

For each rare disease (Kaposi sarcoma, toxoplasmosis, and Kawasaki disease), a cohort of patients was constructed and was compared to each of two control cohorts: one cohort of patients with PTSD and one with influenza (Table 1). The coded data used in the analyses covered years 2004 to 2009 since this time period has the richest and most complete record sets in the hospital. Discharge summary data from 1991 to 2009 were used. Relevant PubMed and Wikipedia articles from any year of publication were analyzed. Table 3 shows a selected list of highly statistically significant associated medical conditions with these rare diseases compared to either or both control cohorts. (A complete list of all significant associations are shown in Tables S1, S2, S3, S4, S5, S6.)

**Table 3:** A selected list of significantly associated diseases with Kaposi sarcoma, toxoplasmosis, and Kawasaki disease, determined from the NYPH EHR, compared against either or both control groups of influenza and PTSD patients.

ICD-9	Description	Odds ratio	P-value	FDR
	**Kaposi sarcoma vs. influenza and PTSD**			
176.0	Kaposi's sarcoma skin	N/A	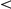 0.001	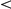 0.001
176.1	Kaposi's sarcoma soft tissue	116.83	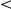 0.001	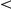 0.001
176.2	Kaposi's sarcoma palate	N/A	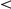 0.001	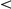 0.001
	**Kaposi sarcoma vs. influenza only**			
110.3	Dermatophytosis of groin and perianal area	15.58	0.001	0.005
110.4	Dermatophytosis of foot	3.46	0.005	0.022
112.2	Candidiasis of other urogenital sites	7.79	0.005	0.023
	**Kaposi sarcoma vs. PTSD only**			
078.5	Cytomegaloviral disease	23.19	0.002	0.007
786.3	Hemoptysis	9.66	0.003	0.012
284.1	Pancytopenia	4.83	0.014	0.044
	**Toxoplasmosis vs. influenza and PTSD**			
136.3	Pneumocystosis	24.95	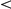 0.001	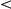 0.001
176.0	Kaposi's sarcoma skin	N/A	0.003	0.022
176.4	Kaposi's sarcoma lung	N/A	0.003	0.023
	**Toxoplasmosis vs. influenza only**			
070.32	Chronic viral hepatitis b without hepatic coma without hepatitis delta	12.47	0.001	0.011
070.54	Chronic hepatitis c without hepatic coma	3.85	0.004	0.029
054.10	Genital herpes unspecified	6.24	0.007	0.041
	**Toxoplasmosis vs. PTSD only**			
038.0	Streptococcal septicemia	13.92	0.008	0.047
038.8	Other specified septicemias	13.92	0.008	0.047
038.19	Other staphylococcal septicemia	N/A	0.009	0.048
	**Kawasaki vs. influenza and PTSD**			
372.30	Conjunctivitis unspecified	3.67	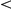 0.001	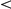 0.001
034.1	Scarlet fever	10.39	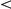 0.001	0.015
299.0	Autistic disorder current or active state	15.15	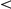 0.001	0.017
	**Kawasaki vs. PTSD only**			
462	Acute pharyngitis	2.28	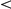 0.001	0.003
446.5	Giant cell arteritis	24.06	0.002	0.020
447.8	Other specified disorders of arteries and arterioles	N/A	0.003	0.034

If there are no patients with a diagnosis code in the control groups, odds ratio is not calculated (i.e. N/A). Kawasaki vs. influenza yields no significant associations not already found in Kawasaki vs. PTSD.

**Figure 3:**
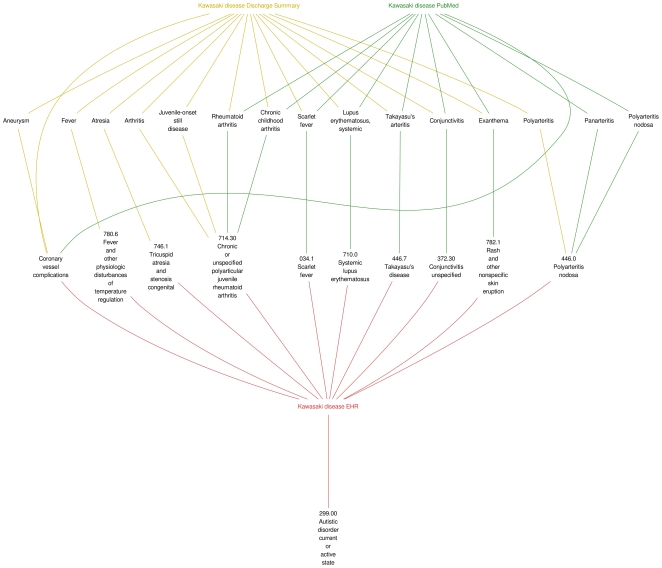
The network of interactions of statistically significant diseases associated with Kawasaki disease compared to influenza combined with results from NLP reports, PubMed articles and Wikipedia articles. Diseases linked to the diagnoses from either PubMed (green links) or Wikipedia (blue links) are documented associations. Diseases associated purely from diagnoses (red links) or NLP reports (gold links) are novel associations that have not been reported before.

Fig. 3 and Fig. S1 and S2 show the results of combining all four data sources for determining significant disease associations compared to influenza. (Similar diagrams obtained with PTSD are not shown.) Associated diseases can be split into two sets: those that confirm known relationships where the results from the diagnoses, PubMed articles, and Wikipedia overlap, and novel associations that occur only in the diagnoses or NLP reports.

The EHR codes associated with a case disease can be divided into the following groups: 1) codes of well-known characteristics of the disease; 2) codes related to well-known disease associations and their manifestations; 3) codes of disease treatments and their sequelae; 4) codes related to treatment of associated diseases and their sequelae; 5) confounded, rare, or previously unreported disease associations.

ADAMS identified a total of 303 ICD-9-CM codes associated with Kaposi sarcoma that were statistically significant across both PTSD and influenza control groups. Of these codes, 178 were shared between both control groups, 82 codes were associated only with influenza, and 43 were associated only with PTSD. A total of 88 codes were different types of specific malignant neoplasm, 19 of which were associated only with flu, whereas only 1 was associated only with PTSD.

The ICD-9-CM codes directly related to Kaposi sarcoma include “176.0 Kaposi sarcoma skin,” “176.3 Kaposi sarcoma gastrointestinal sites,” “176.4 Kaposi sarcoma lung,” and “280.0 Iron deficiency anemia secondary to blood loss (chronic).” Since Kaposi sarcoma is strongly associated with HIV infection, many codes related to HIV and opportunistic infections appear: “117.5 Cryptococcosis,” “136.3 Pneumocystosis,” “031.2 Disseminated mycobacterium.” Liposomal anthracyclines are among the treatments for Kaposi sarcoma [38]. One of their known side effects is cardiotoxicity: “427.89 Other specified cardiac dysrhythmias.” The mainstay treatment of HIV-infected individuals is HAART. Known side effects of HAART include: “250.00 Diabetes mellitus without complication type ii or unspecified type not stated as uncontrolled,” “272.4 Other and unspecified hyperlipidemia,” and “357.6 Polyneuropathy due to drugs.” Kaposi sarcoma is also found to be associated with “282.60 Sickle-cell disease unspecified,” for which there is limited describing literature.

ADAMS identified a total of 162 ICD-9-CM codes associated with toxoplasmosis that were statistically significant across both PTSD and influenza control groups. Of these codes, 86 were shared between both control groups, 23 codes were associated only with influenza, and 53 were associated only with PTSD. ADAMS identified 15 codes associated with toxoplasmosis that were statistically significant compared to Kaposi sarcoma as the control group; and 35 codes associated with Kaposi sarcoma that were statistically significant compared to toxoplasmosis (Table 4 and Table S7).

**Table 4:** Significantly associated diseases with toxoplasmosis, compared to Kaposi sarcoma (FDR
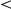
0.05).

ICD-9	Description	Odds ratio	P-value	FDR
130.0	Meningoencephalitis due to toxoplasmosis	70.46	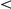 0.001	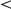 0.001
130.7	Toxoplasmosis of other specified sites	72.07	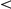 0.001	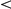 0.001
780.39	Other convulsions	5.52	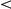 0.001	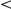 0.001
042	Human immunodeficiency virus (hiv) disease	1.90	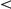 0.001	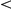 0.001
130.8	Multisystemic disseminated toxoplasmosis	N/A	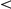 0.001	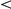 0.001
323.9	Unspecified cause of encephalitis	N/A	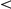 0.001	0.004
345.10	Generalized convulsive epilepsy without intractable epilepsy	16.01	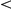 0.001	0.005
345.90	Epilepsy unspecified without intractable epilepsy	3.20	0.001	0.011
130.2	Chorioretinitis due to toxoplasmosis	N/A	0.001	0.012
348.8	Other conditions of brain	N/A	0.001	0.012
784.0	Headache	2.31	0.003	0.021
363.00	Focal chorioretinitis unspecified	N/A	0.003	0.025
364.3	Unspecified iridocyclitis	12.81	0.003	0.025
644.10	Other threatened labor unspecified as to episode of care	12.81	0.003	0.025
648.91	Other current conditions classifiable elsewhere of mother with delivery	6.41	0.009	0.050

Table S7 shows the significantly associated diseases with Kaposi sarcoma, compared to toxoplasmosis. If there are no patients with a diagnosis code in the control groups, odds ratio is not calculated (i.e. N/A).

The ICD-9-CM codes directly related to toxoplasmosis include “130.0 Meningoencophalitis due to toxoplasmosis,” “130.1 Conjunctivitis due to toxoplasmosis,” “130.3 Myocarditis due to toxoplasmosis,” “130.4 Pneumonitis due to toxoplasmosis,” “130.7 Toxoplasmosis of other specified sites,” and “130.8 Multisystemic disseminated toxoplasmosis.” Since toxoplasmosis is strongly associated with HIV infection, many codes related to HIV complications and opportunistic infections appear: “287.5 Leukocytopenia,” “799.4 Cachexia,” “112.84 Candidal esophagitis,” “078.5 Cytomegaloviral disease.” Codes suggestive of peripartum complications and congenital deformities consistent with congenital toxoplasmosis include “130.2 Chorioretinitis due to toxoplasmosis,” “646.83 Other specified antepartum complications,” “648.91 Other current conditions classifiable elsewhere of mother with delivery,” and “655.41 Suspected damage to fetus from other disease in the mother affecting management of mother with delivery.” TMP-SMX is among the first line treatments for toxoplasmosis, and as a sulfa drug it is known to cause exfoliative dermatitis as an adverse drug reaction: “693.0 Dermatitis due to drugs and medicines taken internally.”

ADAMS identified a total of 53 ICD-9-CM codes associated with Kawasaki disease that were statistically significant across both PTSD and influenza control groups. Of these codes, 12 were shared between both control groups, none were associated only with influenza, and 41 were associated only with PTSD.

The ICD-9-CM codes directly related to Kawasaki disease include “372.30 Conjunctivitis unspecified,” “414.11 Aneurysm of coronary vessels,” “780.6 Fever and other physiologic disturbances of temperature regulation,” and “782.1 Rash and other nonspecific skin eruption.” The differential diagnosis of Kawasaki disease would include other infectious and rheumatic diseases, such as “034.1 Scarlet fever,” “446.0 Polyarteritis nodosa,” “446.7 Takayasu's disease,” “710.0 Systemic lupus erythematosus,” and “714.30 Chronic or unspecified polyarticular juvenile rheumatoid arthritis.” Codes more likely to occur as a result of the workup for Kawasaki diseases include “746.1 Tricuspid atresia and stenosis congenital” and “746.85 Coronary artery anomaly congenital.” A code that has not been described extensively is “299.00 Autistic disorder current or active.”

## Discussion

The associations identified by ADAMS mostly describe known characteristics of each disease, including symptoms and manifestations, treatments, and possible confounders. Most interestingly, ADAMS identified associations with limited or no reporting in the existing literature.

Kaposi sarcoma has been seen as a frequent and aggressive disease in AIDS patients [39]. Although the vast majority of the associations for Kaposi sarcoma (Tables S1 and S2) can be explained by associations described in the literature, complications related to neoplasms, HIV infection, risk factors for HIV infection (sexually trasmitted infections, drug abuse), diseases associated with HIV infection, and side effects of neoplasm treatment and highly active antiretroviral therapy (HAART), the results showed Kaposi sarcoma is also possibly associated with sickle cell anemia, for which there has been limited research to confirm the links. This relationship, however, may be a reflection of the biases in the patient population at NYPH.

Toxoplasmosis is usually asymptomatic in immunocompetent hosts, hence its most frequent manifestation is as an opportunistic infection in immunocompromised hosts. Most of the ICD-9-CM codes from the intersection of PTSD and influenza comparisons demonstrate findings that are most consistent with HIV patients (Tables S3 and S4), similar to Kaposi sarcoma above. The intersection also contains codes related to complications during the delivery of babies and to neurotoxoplasmosis, e.g., chorioretinitis, which occurs more often in the setting of congenital infection. The codes do not directly implicate a specific disease as the cause of complications during pregnancy or delivery, but they do indicate congenital complications secondary to diseases of the mother, which would be consistent with toxoplasmosis infection during pregnancy. Because the EHR does not track relationships between patients, it was not possible to tie the children with congenital complications to the mothers who had complicated pregnancies or deliveries.

The ICD-9-CM codes from the comparison of toxoplasmosis to influenza yield more psychiatric disease codes, whereas the codes from the comparison to PTSD have more codes that can be associated with infectious diseases in general. These findings demonstrate the value of using two control groups: the intersection of significant codes shows consistency in the paper's methods; and the codes not in the intersection illustrate the biases inherent in the controls. The influenza control group comparison identifies codes that exclude non-specific signs and symptoms when sick with infectious diseases, and the PTSD control group identifies codes that exclude psychiatric associations. Moreover, life-threatening diseases qualify as traumatic events for the diagnosis of PTSD [40], [41], therefore it can be inferred that PTSD patients are more likely to have neoplasms than influenza patients; neoplasm associations will more likely be statistically insignificant with the PTSD cohort as the control. Otherwise, the majority of the associations for toxoplasmosis can be explained by associations described in the literature, complications related to mass effects in the brain, HIV infection, risk factors for HIV infection (sexually trasmitted infections, drug abuse), diseases associated with HIV infection, and side effects of highly active antiretroviral therapy (HAART).

A comparison of Kaposi sarcoma with toxoplasmosis and vice versa should then identify ICD-9-CM codes specific to each while excluding codes related to HIV infection. This is indeed the case (Table 4 and Table S7). Kaposi sarcoma compared to toxoplasmosis as the control cohort shows neoplasms and other findings specific to Kaposi sarcoma. Toxoplasmosis compared to Kaposi sarcoma as the control cohort shows classical findings of toxoplasmosis. Toxoplasmosis is still associated with the disease code for HIV infection when compared to Kaposi sarcoma. This observation is reasonable given that toxoplasmosis occurs when CD4 counts fall below one hundred cells per cubic millimeter of blood, indicating significant immunocompromise, whereas Kaposi sarcoma can occur in non-HIV-infected individuals. It stands to reason that toxoplasmosis is more dependent on severe immunocompromise in order to manifest, hence its stronger association with HIV infection than Kaposi sarcoma's. [42], [43].

These findings show that choosing a sicker control cohort makes statistical testing less sensitive and more specific, while choosing a less sick cohort does the converse. A sicker control cohort would have greater counts of patients with ICD-9-CM codes related to that disease than a less sick cohort of the same disease. For those codes to be statistically significant associations with the case disease, the case cohort would have to have even larger counts of patients with the same ICD-9-CM codes; or the control cohort would have to be larger with the same proportion of patients with the same ICD-9-CM codes. It then becomes necessary to choose control cohorts carefully in order to be aware of their inherent biases since these biases directly affect the results and guide interpretation. Thus using multiple control cohorts whose biases are known can provide richer results and interpretations than using control cohorts that are created by random sampling of patients where biases are more uncertain.

In the case of Kawasaki disease, ADAMS identified codes that reflected the known signs and symptoms of the disease (Tables S5 and S6). The disease codes found by comparing to influenza were a complete subset of those found by comparing to PTSD. The codes are highly specific to Kawasaki disease and demonstrate associations with other autoimmune diseases. The remaining codes found by comparing to PTSD represent non-specific findings of sickness from infectious etiology.

Among the intersection of significant ICD-9-CM codes were scarlet fever, juvenile rheumatoid arthritis, Takayasu disease, and systemic lupus erythematosus. Scarlet fever is part of the differential diagnosis since both scarlet fever and Kawasaki disease present with strawberry tongue and rash [44], [45]. The others are rheumatological diseases that can present with similar symptoms [46]–[48]. Were the coded data for a longer longitudinal period, the associations could be observed as diseases that occurred in the same individuals later in life, thus changing the interpretation from differential diagnosis to disease association. The statistically significant associations with “746.1 Tricuspid atresia and stenosis congenital” and “746.85 Coronary artery anomaly congenital” reflect bias from the workup for Kawasaki disease, which includes echocardiography to establish a baseline for possible coronary artery aneurysm.

Most importantly, the statistically significant association with “299.00 Autistic disorder current or active” state may reflect a sequela from the systemic inflammation of Kawasaki disease that thus far has not been well described. A case study by Tabarki *et. al.* [49] suggested a possible link between Kawasaki disease and autistic behavior for which we report a statistical evidence of an association at NYPH, which has a much larger patient cohort. Definitive neurological complications of Kawasaki disease have been described but are limited to the acute setting. Long-term complications of autism as a result of Kawasaki disease or autistic individuals being more susceptible to Kawasaki disease have not been described extensively. There is literature on the controversy of whether autism and Kawasaki disease are complications of vaccines [50]–[54]. It is possible that the inflammatory response that underlies Kawasaki disease may effect neurological changes that result in autism, or the susceptibility to the underlying pathophysiology may lead to both diseases occurring independently in the same individual. The limitation of the current study of ADAMS does not evaluate the time course of disease. Further study of the clinical histories of the patients with both Kawasaki disease and autism as identified by ADAMS merits further pursuit. With respect to the network diagrams for Kawasaki disease, it should be noted that there are no connections from external sources to autism despite the Tabarki case report. The lack of connections occurred because autism was described only in the body of the paper and not in the abstract; natural language processing was done only on abstracts.

The NYPH EHR suffers from data biases. First, there are multiple ways that information can be entered into the system, and each department within the hospital can follow its own set of guidelines for data entry. Second, NYPH mainly serves patients in Manhattan and to a lesser extent the other four boroughs, so its EHR is mostly representative of the population of New York City. While we can draw conclusions of clinical interest from this population, they may not necessarily represent disease associations at a national or global level.

ADAMS is by current design ICD-9-CM centric. It takes knowledge from coded hospital records and corroborates the findings with other data sources. Therefore, it should be noted that there may be links between medical conditions that ADAMS cannot identify because those conditions lack coded descriptions. Future work will be focused on analyzing the relationships for diseases that have no clear ICD-9-CM codes, especially using textual reports that have undergone natural language processing.

ADAMS can be applied to the analysis of less rare diseases. It would find more associations, since more common diseases would have larger cohorts and thus be more likely to have different comorbidities across all the individuals. To cope with the larger number of associations, the FDR cutoff can be set to a lower threshold in order to reduce the number of statistically significant findings. Otherwise, investigators with clinical expertise will have to spend more time analyzing the longer list to rule out confounding. This may still be fruitful, albeit time consuming, since there may be true associations that would be excluded by a stricter FDR cutoff. By focusing on rare diseases in this paper, the burden of many associations and confounding was intrinsically limited, which made analysis and interpretation easier and more manageable.

In conclusion, ADAMS confirms relationships between these three rare diseases and other medical conditions that have already been reported in PubMed or Wikipedia. This means our method is capable of detecting signal amid the noise in the NYPH EHR with greater confidence than simply using statistical multiple hypothesis testing methods. In particular, the starting points for further investigations are the results reported only in the EHR that have not been previously described in the literature. Although ADAMS by itself is not a diagnosis tool, we suggest that it could be further refined into a tool for health professionals as an effective means to confirm descriptions in existing literature and to identify under-recognized or undiscovered associations for further clinical inquiry.

## Supporting Information

Figure S1 **The network of interactions of statistically significant diseases associated with Kaposi sarcoma compared to influenza combined with results from NLP reports, PubMed articles and Wikipedia articles.** Diseases linked to the diagnoses from either PubMed (green links) or Wikipedia (blue links) are documented associations. Diseases associated purely from diagnoses (red links) or NLP reports (gold links) are novel associations that have not been reported before. Here, we have used custom categories feature (Methods) to aid clarity.
(PDF)

Figure S2 **The network of interactions of statistically significant diseases associated with toxoplasmosis compared to influenza combined with results from NLP reports, PubMed articles and Wikipedia articles.** Diseases linked to the diagnoses from either PubMed (green links) or Wikipedia (blue links) are documented associations. Diseases associated purely from diagnoses (red links) or NLP reports (gold links) are novel associations that have not been reported before.
(PDF)

Figure S3 **The interface of ADAMS application, where the case and control cohorts are selected. ADAMS is also able to limit time intervals for data. The desired number of bootstraps is also entered here.** Clicking on the “Search” button in ADAMS begins the process of statistical analysis.
(PDF)

Table S1
**Significantly associated diseases with Kaposi sarcoma, compared to the influenza control cohort (FDR** 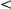 **0.05).** If there are no patients with a diagnosis code in the control groups, odds ratio is not calculated (i.e. N/A).(PDF)

Table S2
**Significantly associated diseases with Kaposi sarcoma, compared to the PTSD control cohort (FDR** 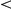 **0.05).** If there are no patients with a diagnosis code in the control groups, odds ratio is not calculated (i.e. N/A).(PDF)

Table S3
**Significantly associated diseases with toxoplasmosis, compared to the influenza control cohort (FDR** 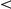 **0.05).** If there are no patients with a diagnosis code in the control groups, odds ratio is not calculated (i.e. N/A).(PDF)

Table S4
**Significantly associated diseases with toxoplasmosis, compared to the PTSD control cohort (FDR** 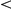 **0.05).** If there are no patients with a diagnosis code in the control groups, odds ratio is not calculated (i.e. N/A).(PDF)

Table S5
**Significantly associated diseases with Kawasaki sdisease, compared to the influenza control cohort (FDR** 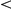 **0.05).** If there are no patients with a diagnosis code in the control groups, odds ratio is not calculated (i.e. N/A).(PDF)

Table S6
**Significantly associated diseases with Kawasaki disease, compared to the PTSD control cohort (FDR** 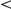 **0.05).** If there are no patients with a diagnosis code in the control groups, odds ratio is not calculated (i.e. N/A).(PDF)

Table S7
**Significantly associated diseases with Kaposi sarcoma, compared to toxoplasmosis control cohort (FDR** 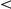 **0.05).** If there are no patients with a diagnosis code in the control groups, odds ratio is not calculated (i.e. N/A).(PDF)
