# Supplementary material for: Discovering Disease Associations by Integrating Electronic Clinical Data and Medical Literature
Source: PLoS One. 2011 Jun 23;6(6):e21132. doi: 10.1371/journal.pone.0021132 (PMC3121722; doi:10.1371/journal.pone.0021132)
Supplement: Figure S2 — The network of interactions of statistically significant diseases associated with toxoplasmosis compared to influenza combined with results from NLP reports, PubMed articles and Wikipedia articles. Diseases linked to the diagnoses from either PubMed (green links) or Wikipedia (blue links) are documented associations. Diseases associated purely from diagnoses (red links) or NLP reports (gold links) are novel associations that have not been reported before. (PDF) [file pone.0021132.s002.pdf]

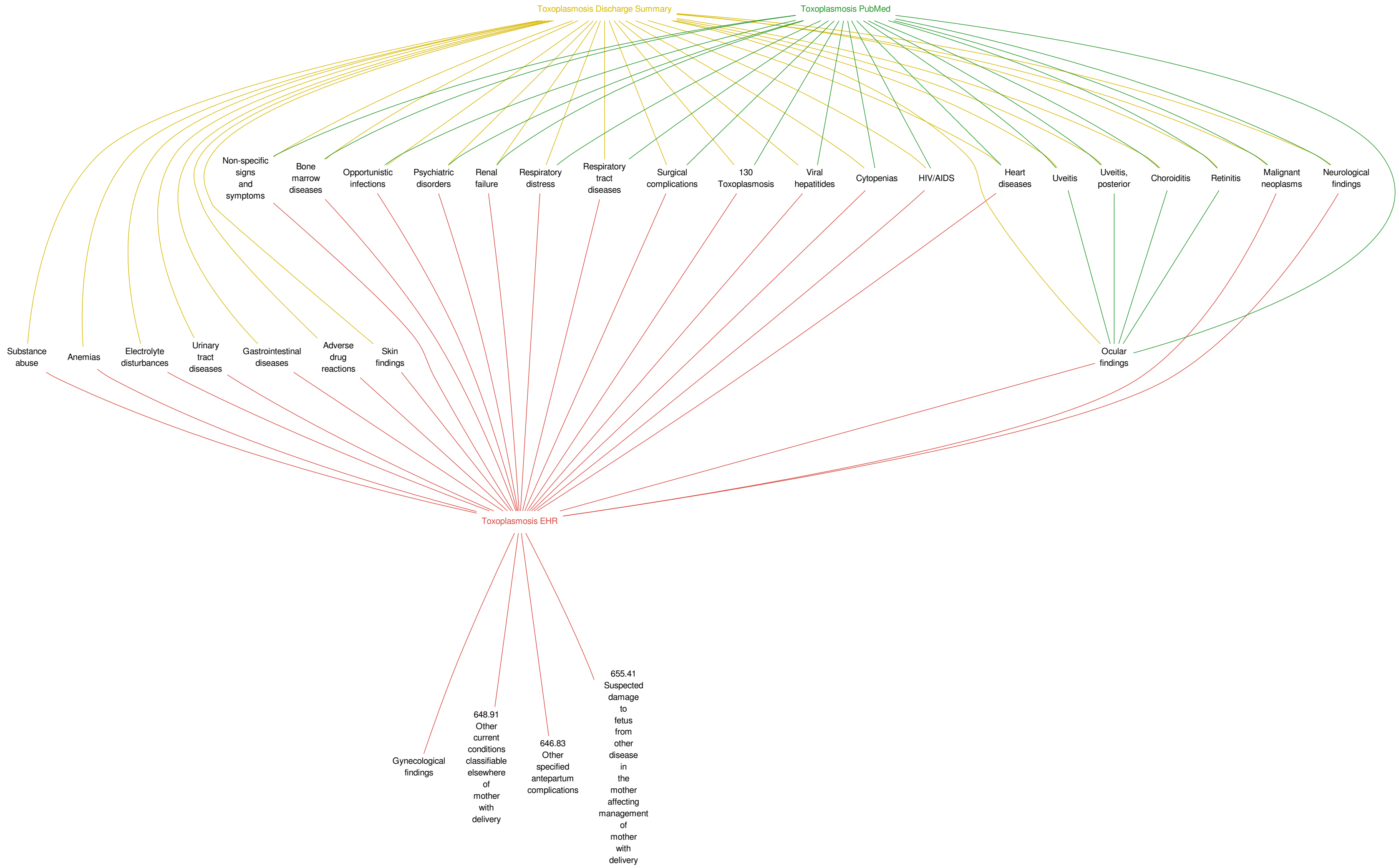

Supporting Figure S2 . The network of interactions of statistically significant diseases associated with toxoplasmosis compared to influenza combined with results from NLP reports, PubMed articles and Wikipedia articles. Diseases linked to the diagnoses from either PubMed (green links) or Wikipedia (blue links) are documented associations. Diseases associated purely from diagnoses (red links) or NLP reports (gold links) are novel associations that have not been reported before.
