# Supplementary material for: Discovering Disease Associations by Integrating Electronic Clinical Data and Medical Literature
Source: PLoS One. 2011 Jun 23;6(6):e21132. doi: 10.1371/journal.pone.0021132 (PMC3121722; doi:10.1371/journal.pone.0021132)
Supplement: Figure S3 — The interface of ADAMS application, where the case and control cohorts are selected. ADAMS is also able to limit time intervals for data. The desired number of bootstraps is also entered here. Clicking on the “Search” button in ADAMS begins the process of statistical analysis. (PDF) [file pone.0021132.s003.pdf]

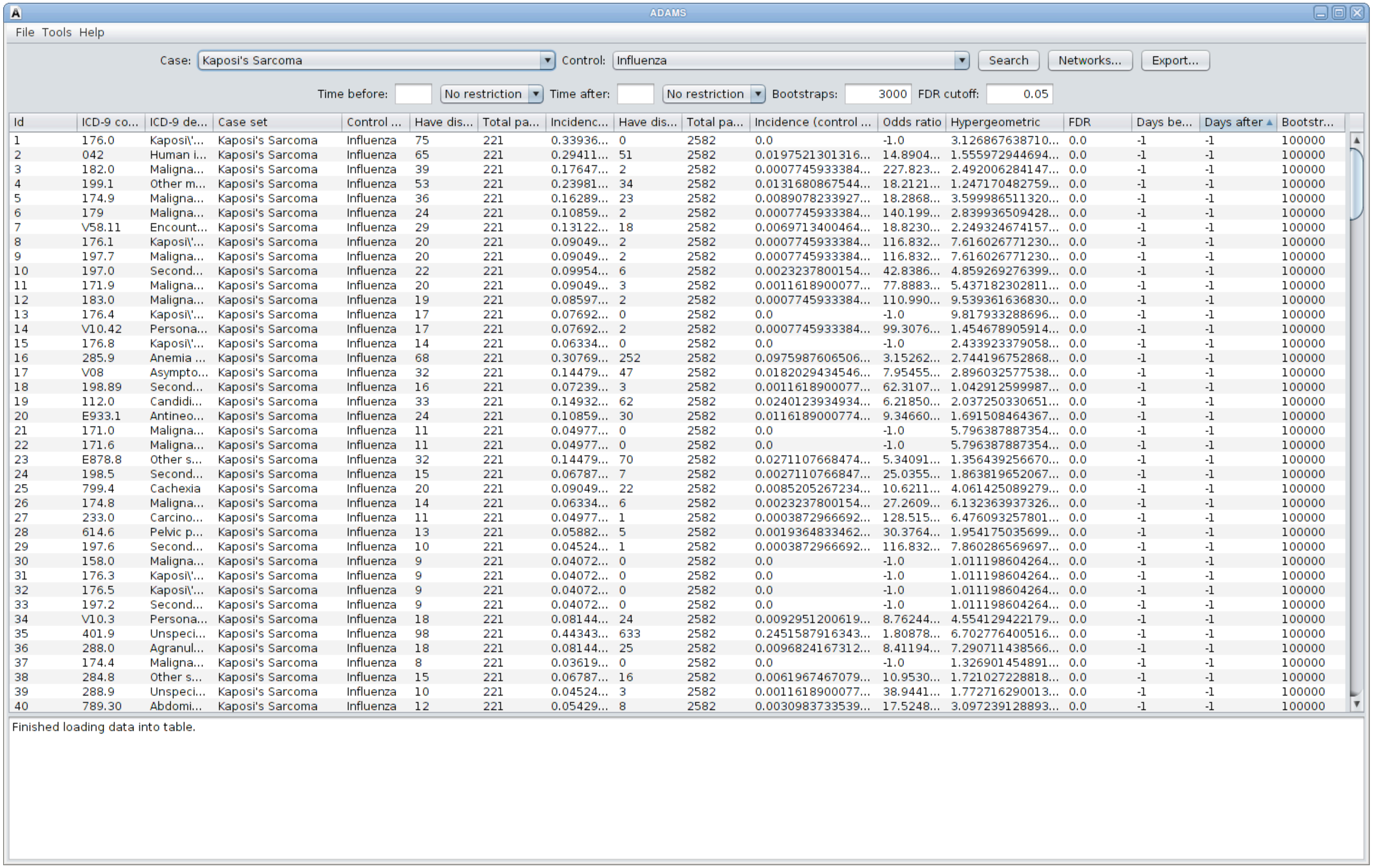

Supporting Figure S3 . The interface of ADAMS application, where the case and control cohorts are selected. ADAMS is also able to limit time intervals for data. The desired number of bootstraps is also entered here. Clicking on the "Search" button in ADAMS begins the process of statistical analysis.
