# Supplementary material for: Discovering Disease Associations by Integrating Electronic Clinical Data and Medical Literature
Source: PLoS One. 2011 Jun 23;6(6):e21132. doi: 10.1371/journal.pone.0021132 (PMC3121722; doi:10.1371/journal.pone.0021132)
Supplement: Table S1 — Significantly associated diseases with Kaposi sarcoma, compared to the influenza control cohort (FDR 0.05). If there are no patients with a diagnosis code in the control groups, odds ratio is not calculated (i.e. N/A). (PDF) [file pone.0021132.s004.pdf]

Supporting Table S1 . Significantly associated diseases with Kaposi sarcoma, compared to the influenza control cohort (FDR < 0.05). If there are no patients with a diagnosis code in the control groups, odds ratio is not calculated (i.e. N/A).

ICD-9	Description	Odds ratio	P-value	FDR
042	Human immunodeficiency virus (hiv) disease	14.89	<0.001	<0.001
112.0	Candidiasis of mouth	6.22	<0.001	<0.001
136.3	Pneumocystosis	21.42	<0.001	<0.001
158.0	Malignant neoplasm of retroperitoneum	N/A	<0.001	<0.001
171.0	Malignant neoplasm of connective and other soft tissue of head face and neck	N/A	<0.001	<0.001
171.6	Malignant neoplasm of connective and other soft tissue of pelvis	N/A	<0.001	<0.001
171.9	Malignant neoplasm of connective and other soft tissue site unspecified	77.89	<0.001	<0.001
173.8	Other malignant neoplasm of other specified sites of skin	93.47	<0.001	<0.001
174.4	Malignant neoplasm of upper-outer quadrant of female breast	N/A	<0.001	<0.001
174.8	Malignant neoplasm of other specified sites of female breast	27.26	<0.001	<0.001
174.9	Malignant neoplasm of breast (female) unspecified site	18.29	<0.001	<0.001
176.0	Kaposi's sarcoma skin	N/A	<0.001	<0.001
176.1	Kaposi's sarcoma soft tissue	116.83	<0.001	<0.001
176.3	Kaposi's sarcoma gastrointestinal sites	N/A	<0.001	<0.001
176.4	Kaposi's sarcoma lung	N/A	<0.001	<0.001
176.5	Kaposi's sarcoma lymph nodes	N/A	<0.001	<0.001
176.8	Kaposi's sarcoma other specified sites	N/A	<0.001	<0.001
179	Malignant neoplasm of uterus-part unspecified	140.20	<0.001	<0.001
182.0	Malignant neoplasm of corpus uteri except isthmus	227.82	<0.001	<0.001
183.0	Malignant neoplasm of ovary	110.99	<0.001	<0.001
197.0	Secondary malignant neoplasm of lung	42.84	<0.001	<0.001
197.2	Secondary malignant neoplasm of pleura	N/A	<0.001	<0.001
197.6	Secondary malignant neoplasm of retroperitoneum and peritoneum	116.83	<0.001	<0.001
197.7	Malignant neoplasm of liver secondary	116.83	<0.001	<0.001
198.5	Secondary malignant neoplasm of bone and bone marrow	25.04	<0.001	<0.001
198.89	Secondary malignant neoplasm of other specified sites	62.31	<0.001	<0.001
199.1	Other malignant neoplasm of unspecified site	18.21	<0.001	<0.001
200.18	Lymphosarcoma involving lymph nodes of multiple sites	93.47	<0.001	<0.001
233.0	Carcinoma in situ of breast	128.52	<0.001	<0.001
284.8	Other specified aplastic anemias	10.95	<0.001	<0.001
285.1	Acute posthemorrhagic anemia	8.50	<0.001	<0.001
285.9	Anemia unspecified	3.15	<0.001	<0.001
288.0	Agranulocytosis	8.41	<0.001	<0.001
288.9	Unspecified disease of white blood cells	38.94	<0.001	<0.001
401.9	Unspecified essential hypertension	1.81	<0.001	<0.001
614.6	Pelvic peritoneal adhesions female (postoperative) (postinfection)	30.38	<0.001	<0.001
789.30	Abdominal or pelvic swelling mass or lump unspecified site	17.52	<0.001	<0.001
799.4	Cachexia	10.62	<0.001	<0.001
584.9	Acute renal failure unspecified	3.00	<0.001	<0.001
182.8	Malignant neoplasm of other specified sites of body of uterus	N/A	<0.001	<0.001
070.30	Viral hepatitis b without hepatic coma acute or unspecified without hepatitis delta	21.03	<0.001	<0.001
282.60	Sickle-cell disease unspecified	5.05	<0.001	<0.001
159.0	Malignant neoplasm of intestinal tract part unspecified	N/A	<0.001	<0.001
176.2	Kaposi's sarcoma palate	N/A	<0.001	<0.001
196.3	Secondary and unspecified malignant neoplasm of lymph nodes of axilla and upper limb	N/A	<0.001	<0.001
451.11	Phlebitis and thrombophlebitis of femoral vein (deep) (superficial)	17.52	<0.001	<0.001
287.5	Thrombocytopenia unspecified	3.41	<0.001	<0.001
262	Other severe protein-calorie malnutrition	9.89	<0.001	<0.001

Continued on next page

Supporting Table S1 – continued from previous page

ICD-9	Description	Odds ratio	P-value	FDR
174.5	Malignant neoplasm of lower-outer quadrant of female breast	40.89	<0.001	<0.001
180.9	Malignant neoplasm of cervix uteri unspecified site	40.89	<0.001	<0.001
611.8	Other specified disorders of breast	40.89	<0.001	<0.001
284.89	Red cell aplasia (acquired)(adult) (with thymoma)	15.02	<0.001	<0.001
998.59	Other postoperative infection	6.90	<0.001	<0.001
162.9	Malignant neoplasm of bronchus and lung unspecified	10.62	<0.001	<0.001
171.8	Malignant neoplasm of other specified sites of connective and other soft tissue	70.10	<0.001	<0.001
174.0	Malignant neoplasm of nipple and areola of female breast	70.10	<0.001	<0.001
198.82	Secondary malignant neoplasm of genital organs	70.10	<0.001	<0.001
288.00	Neutropenia, unspecified	5.84	<0.001	<0.001
159.9	Malignant neoplasm of ill-defined sites within the digestive organs and peritoneum	N/A	<0.001	<0.001
171.3	Malignant neoplasm of connective and other soft tissue of lower limb including hip	N/A	<0.001	<0.001
171.4	Malignant neoplasm of connective and other soft tissue of thorax	N/A	<0.001	<0.001
180.8	Malignant neoplasm of other specified sites of cervix	N/A	<0.001	<0.001
197.4	Secondary malignant neoplasm of small intestine including duodenum	N/A	<0.001	<0.001
285.22	Anemia in neoplastic disease	10.51	<0.001	<0.001
031.2	Disseminated mycobacterium	35.05	<0.001	<0.001
707.03	Chronic ulcer of skin, lower back	7.14	<0.001	<0.001
611.72	Lump or mass in breast	3.82	<0.001	<0.001
276.1	Hyposmolality and/or hyponatremia	3.44	<0.001	<0.001
218.1	Intramural leiomyoma of uterus	16.36	<0.001	<0.001
453.41	Venous embolism and thrombosis of deep vessels of proximal lower extremity	16.36	<0.001	<0.001
356.9	Unspecified idiopathic peripheral neuropathy	10.39	<0.001	<0.001
296.80	Bipolar disorder, unspecified	6.12	<0.001	<0.001
620.2	Other and unspecified ovarian cyst	6.12	<0.001	<0.001
682.2	Cellulitis and abscess of trunk	6.12	<0.001	<0.001
155.2	Malignant neoplasm of liver not specified as primary or secondary	58.42	<0.001	<0.001
170.9	Malignant neoplasm of bone and articular cartilage site unspecified	58.42	<0.001	<0.001
199.0	Disseminated malignant neoplasm	58.42	<0.001	<0.001
453.8	Embolism and thrombosis of other specified veins	7.51	<0.001	<0.001
238.0	Neoplasm of uncertain behavior of bone and articular cartilage	17.52	<0.001	<0.001
305.60	Nondependent cocaine abuse unspecified use	4.47	<0.001	<0.001
158.8	Malignant neoplasm of specified parts of peritoneum	N/A	<0.001	<0.001
158.9	Malignant neoplasm of peritoneum unspecified	N/A	<0.001	<0.001
170.6	Malignant neoplasm of pelvic bones sacrum and coccyx	N/A	<0.001	<0.001
184.0	Malignant neoplasm of vagina	N/A	<0.001	<0.001
198.0	Secondary malignant neoplasm of kidney	N/A	<0.001	<0.001
198.81	Secondary malignant neoplasm of breast	N/A	<0.001	<0.001
614.1	Chronic salpingitis and oophoritis	N/A	<0.001	<0.001
785.6	Enlargement of lymph nodes	4.34	<0.001	<0.001
220	Benign neoplasm of ovary	29.21	<0.001	<0.001
191.9	Malignant neoplasm of brain unspecified site	9.09	<0.001	<0.001
356.4	Idiopathic progressive polyneuropathy	19.47	<0.001	<0.001
185	Malignant neoplasm of prostate	5.84	<0.001	<0.001
070.54	Chronic hepatitis c without hepatic coma	4.12	<0.001	<0.001
366.10	Senile cataract unspecified	3.17	<0.001	<0.001
285.29	Anemia of other chronic illness	4.01	<0.001	0.001
707.9	Chronic ulcer of unspecified site	4.87	<0.001	0.001
171.5	Malignant neoplasm of connective and other soft tissue of abdomen	46.73	<0.001	0.001
180.0	Malignant neoplasm of endocervix	46.73	<0.001	0.001
196.9	Secondary and unspecified malignant neoplasm of lymph nodes site unspecified	46.73	<0.001	0.001
453.42	Venous embolism and thrombosis of deep vessels of distal lower extremity	46.73	<0.001	0.001
623.8	Other specified noninflammatory disorders of vagina	3.41	<0.001	0.001
627.1	Postmenopausal bleeding	7.43	<0.001	0.002
682.6	Cellulitis and abscess of leg except foot	3.34	<0.001	0.002
783.21	Loss of weight	3.79	<0.001	0.002

Continued on next page

Supporting Table S1 – continued from previous page

ICD-9	Description	Odds ratio	P-value	FDR
157.9	Malignant neoplasm of pancreas part unspecified	14.60	<0.001	0.002
202.90	Other and unspecified malignant neoplasms of lymphoid and histiocytic tissue unspecified site	14.60	<0.001	0.002
998.11	Hemorrhage complicating a procedure	5.01	<0.001	0.002
151.9	Malignant neoplasm of stomach unspecified site	23.37	<0.001	0.002
218.2	Subserous leiomyoma of uterus	23.37	<0.001	0.002
305.1	Nondependent tobacco use disorder	2.24	<0.001	0.002
585.9	Chronic kidney disease, unspecified	2.41	<0.001	0.002
117.5	Cryptococcosis	N/A	<0.001	0.002
172.7	Malignant melanoma of skin of lower limb including hip	N/A	<0.001	0.002
172.8	Malignant melanoma of other specified sites of skin	N/A	<0.001	0.002
183.4	Malignant neoplasm of parametrium	N/A	<0.001	0.002
183.9	Malignant neoplasm of uterine adnexa unspecified site	N/A	<0.001	0.002
198.1	Secondary malignant neoplasm of other urinary organs	N/A	<0.001	0.002
198.6	Secondary malignant neoplasm of ovary	N/A	<0.001	0.002
198.7	Secondary malignant neoplasm of adrenal gland	N/A	<0.001	0.002
357.6	Polyneuropathy due to drugs	N/A	<0.001	0.002
453.40	Venous embolism and thrombosis of unspecified deep vessels of lower extremity	11.68	<0.001	0.003
238.2	Neoplasm of uncertain behavior of skin	7.79	<0.001	0.003
562.10	Diverticulosis of colon (without hemorrhage)	2.56	<0.001	0.003
415.19	Other pulmonary embolism and infarction	5.19	<0.001	0.003
375.15	Tear film insufficiency unspecified	4.38	<0.001	0.004
783.7	Adult failure to thrive	4.38	<0.001	0.004
112.84	Candidal esophagitis	5.84	<0.001	0.004
070.70	Unspecified viral hepatitis c without hepatic coma	4.92	<0.001	0.004
998.89	Other specified complications of procedures not elsewhere classified	7.01	<0.001	0.004
571.5	Cirrhosis of liver without alcohol	3.77	<0.001	0.005
110.3	Dermatophytosis of groin and perianal area	15.58	0.001	0.005
793.4	Nonspecific abnormal findings on radiological and other examination of gastrointestinal tract	15.58	0.001	0.005
272.4	Other and unspecified hyperlipidemia	1.91	0.001	0.006
599.7	Hematuria	2.77	0.001	0.006
569.0	Anal and rectal polyp	6.37	0.001	0.006
586	Renal failure unspecified	6.37	0.001	0.006
053.9	Herpes zoster without complication	5.11	0.001	0.006
707.15	Ulcer of other part of foot	5.11	0.001	0.006
218.0	Submucous leiomyoma of uterus	8.35	0.001	0.006
239.0	Neoplasm of unspecified nature of digestive system	8.35	0.001	0.006
153.8	Malignant neoplasm of other specified sites of large intestine	35.05	0.002	0.007
172.9	Melanoma of skin site unspecified	35.05	0.002	0.007
174.3	Malignant neoplasm of lower-inner quadrant of female breast	35.05	0.002	0.007
191.0	Malignant neoplasm of cerebrum except lobes and ventricles	35.05	0.002	0.007
195.2	Malignant neoplasm of abdomen	35.05	0.002	0.007
196.5	Secondary and unspecified malignant neoplasm of lymph nodes of inguinal region and lower limb	35.05	0.002	0.007
200.10	Lymphosarcoma unspecified site	35.05	0.002	0.007
336.3	Myelopathy in other diseases classified elsewhere	35.05	0.002	0.007
587	Renal sclerosis unspecified	35.05	0.002	0.007
789.33	Abdominal or pelvic swelling mass or lump right lower quadrant	35.05	0.002	0.007
305.61	Nondependent cocaine abuse continuous use	4.81	0.002	0.010
789.5	Ascites	4.81	0.002	0.010
782.3	Edema	3.13	0.002	0.010
455.0	Internal hemorrhoids without complication	2.54	0.002	0.010
528.9	Other and unspecified diseases of the oral soft tissues	7.30	0.002	0.012
789.07	Abdominal pain generalized	3.25	0.002	0.012
070.9	Unspecified viral hepatitis without hepatic coma	5.39	0.003	0.012
286.9	Other and unspecified coagulation defects	4.54	0.002	0.012
218.9	Leiomyoma of uterus unspecified	3.89	0.003	0.013
311	Depressive disorder not elsewhere classified	1.63	0.003	0.013
998.12	Hematoma complicating a procedure	6.49	0.003	0.014
458.0	Orthostatic hypotension	5.01	0.003	0.015
585	Chronic kidney disease (ckd)	3.07	0.003	0.015
305.62	Nondependent cocaine abuse episodic use	9.35	0.003	0.015

Continued on next page

Supporting Table S1 – continued from previous page

ICD-9	Description	Odds ratio	P-value	FDR
995.91	Systemic inflammatory response syndrome due to infectious process without organ dysfunction	2.70	0.004	0.016
250.00	Diabetes mellitus without complication type ii or unspecified type not stated as uncontrolled	1.60	0.004	0.017
229.9	Benign neoplasm of unspecified site	4.09	0.004	0.017
188.9	Malignant neoplasm of bladder part unspecified	17.52	0.004	0.017
196.8	Secondary and unspecified malignant neoplasm of lymph nodes of multiple sites	17.52	0.004	0.017
336.9	Unspecified disease of spinal cord	17.52	0.004	0.017
593.4	Other ureteric obstruction	17.52	0.004	0.017
611.2	Fissure of nipple	17.52	0.004	0.017
998.51	Infected postoperative seroma	17.52	0.004	0.017
511.9	Unspecified pleural effusion	2.31	0.004	0.017
793.80	Unspecified abnormal mammogram	2.60	0.005	0.022
153.9	Malignant neoplasm of colon unspecified site	5.84	0.005	0.022
568.0	Peritoneal adhesions (postoperative) (postinfection)	5.84	0.005	0.022
110.4	Dermatophytosis of foot	3.46	0.005	0.022
518.81	Acute respiratory failure	1.98	0.005	0.023
682.3	Cellulitis and abscess of upper arm and forearm	3.89	0.005	0.023
070.32	Chronic viral hepatitis b without hepatic coma without hepatitis delta	7.79	0.005	0.023
112.2	Candidiasis of other urogenital sites	7.79	0.005	0.023
217	Benign neoplasm of breast	7.79	0.005	0.023
569.49	Other specified disorders of rectum and anus	7.79	0.005	0.023
686.1	Pyogenic granuloma of skin and subcutaneous tissue	7.79	0.005	0.023
698.3	Lichenification and lichen simplex chronicus	7.79	0.005	0.023
455.6	Unspecified hemorrhoids without complication	2.78	0.006	0.023
569.3	Hemorrhage of rectum and anus	2.85	0.005	0.024
008.45	Intestinal infection due to clostridium difficile	2.92	0.007	0.024
785.52	Septic shock	2.72	0.007	0.024
303.91	Other and unspecified alcohol dependence continuous drinking behavior	4.38	0.006	0.024
281.9	Unspecified deficiency anemia	4.12	0.007	0.024
518.89	Other diseases of lung not elsewhere classified	2.50	0.006	0.025
427.89	Other specified cardiac dysrhythmias	2.14	0.008	0.025
159.8	Malignant neoplasm of other sites of digestive system and intra-abdominal organs	N/A	0.006	0.026
170.0	Malignant neoplasm of bones of skull and face except mandible	N/A	0.006	0.026
170.3	Malignant neoplasm of ribs sternum and clavicle	N/A	0.006	0.026
172.6	Malignant melanoma of skin of upper limb including shoulder	N/A	0.006	0.026
173.3	Other malignant neoplasm of skin of other and unspecified parts of face	N/A	0.006	0.026
173.6	Other malignant neoplasm of skin of upper limb including shoulder	N/A	0.006	0.026
174.2	Malignant neoplasm of upper-inner quadrant of female breast	N/A	0.006	0.026
183.2	Malignant neoplasm of fallopian tube	N/A	0.006	0.026
183.8	Malignant neoplasm of other specified sites of uterine adnexa	N/A	0.006	0.026
192.9	Malignant neoplasm of nervous system part unspecified	N/A	0.006	0.026
195.3	Malignant neoplasm of pelvis	N/A	0.006	0.026
196.0	Secondary and unspecified malignant neoplasm of lymph nodes of head face and neck	N/A	0.006	0.026
196.6	Secondary and unspecified malignant neoplasm of intrapelvic lymph nodes	N/A	0.006	0.026
197.5	Secondary malignant neoplasm of large intestine and rectum	N/A	0.006	0.026
197.8	Secondary malignant neoplasm of other digestive organs and spleen	N/A	0.006	0.026
269.9	Unspecified nutritional deficiency	N/A	0.006	0.026
302.85	Gender identity disorder in adolescents or adults	N/A	0.006	0.026
304.23	Cocaine dependence in remission	N/A	0.006	0.026
305.41	Nondependent sedative, hypnotic or anxiolytic abuse continuous use	N/A	0.006	0.026
320.9	Meningitis due to unspecified bacterium	N/A	0.006	0.026
321.0	Cryptococcal meningitis	N/A	0.006	0.026
441.00	Dissection of aorta aneurysm unspecified site	N/A	0.006	0.026

Continued on next page

Supporting Table S1 – continued from previous page

ICD-9	Description	Odds ratio	P-value	FDR
705.83	Hidradenitis	N/A	0.006	0.026
885.0	Traumatic amputation of thumb (complete)(partial) without complication	N/A	0.006	0.026
996.69	Infection and inflammatory reaction due to other internal prosthetic device implant and graft	N/A	0.006	0.026
078.11	Condyloma acuminatum	6.68	0.008	0.027
135	Sarcoidosis	6.68	0.008	0.027
263.8	Other protein-calorie malnutrition	6.68	0.008	0.027
424.90	Endocarditis valve unspecified cause	6.68	0.008	0.027
198.3	Secondary malignant neoplasm of brain and spinal cord	11.68	0.008	0.031
202.83	Other malignant lymphomas involving intra-abdominal lymph nodes	11.68	0.008	0.031
261	Nutritional marasmus	11.68	0.008	0.031
583.9	Nephritis and nephropathy not specified as acute or chronic with unspecified pathological lesion in kidney	11.68	0.008	0.031
584.8	Acute renal failure with other specified pathological lesion in kidney	11.68	0.008	0.031
599.89	Other specified disorders of urinary tract	11.68	0.008	0.031
610.2	Fibroadenosis of breast	11.68	0.008	0.031
578.9	Hemorrhage of gastrointestinal tract unspecified	2.27	0.009	0.031
362.9	Unspecified retinal disorder	3.41	0.009	0.031
038.9	Unspecified septicemia	1.99	0.009	0.032
276.50	"Volume depletion, unspecified"	3.27	0.011	0.033
576.1	Cholangitis	4.49	0.011	0.033
211.3	Benign neoplasm of colon	2.12	0.011	0.034
189.0	Malignant neoplasm of kidney except pelvis	5.84	0.011	0.038
281.1	Other vitamin b12 deficiency anemia	5.84	0.011	0.038
357.9	Unspecified inflammatory and toxic neuropathies	5.84	0.011	0.038
616.0	Cervicitis and endocervicitis	5.84	0.011	0.038
787.99	Other symptoms involving digestive system	5.84	0.011	0.038
492.8	Other emphysema	3.15	0.012	0.038
070.1	Viral hepatitis a without hepatic coma	8.76	0.013	0.038
157.0	Malignant neoplasm of head of pancreas	8.76	0.013	0.038
289.81	Primary hypercoagulable state	8.76	0.013	0.038
292.84	Drug-induced mood disorder	8.76	0.013	0.038
337.1	Peripheral autonomic neuropathy in disorders classified elsewhere	8.76	0.013	0.038
429.89	Other ill-defined heart diseases	8.76	0.013	0.038
569.42	Anal or rectal pain	8.76	0.013	0.038
697.0	Lichen planus	8.76	0.013	0.038
780.79	Other malaise and fatigue	1.78	0.013	0.039
518.0	Pulmonary collapse	2.04	0.014	0.044
790.7	Bacteremia	2.11	0.014	0.044
275.42	Hypercalcemia	4.17	0.014	0.044
621.0	Polyp of corpus uteri	4.17	0.014	0.044
280.0	Iron deficiency anemia secondary to blood loss (chronic)	3.03	0.015	0.044
593.9	Unspecified disorder of kidney and ureter	2.25	0.016	0.047
782.0	Disturbance of skin sensation	2.38	0.015	0.047
296.90	Unspecified episodic mood disorder	3.34	0.016	0.048
366.16	Senile nuclear sclerosis	3.34	0.016	0.048
