# Supplementary material for: Discovering Disease Associations by Integrating Electronic Clinical Data and Medical Literature
Source: PLoS One. 2011 Jun 23;6(6):e21132. doi: 10.1371/journal.pone.0021132 (PMC3121722; doi:10.1371/journal.pone.0021132)
Supplement: Table S2 — Significantly associated diseases with Kaposi sarcoma, compared to the PTSD control cohort (FDR 0.05). If there are no patients with a diagnosis code in the control groups, odds ratio is not calculated (i.e. N/A). (PDF) [file pone.0021132.s005.pdf]

Supporting Table S2 . Significantly associated diseases with Kaposi sarcoma, compared to the PTSD control cohort (FDR < 0.05). If there are no patients with a diagnosis code in the control groups, odds ratio is not calculated (i.e. N/A).

ICD-9	Description	Odds ratio	P-value	FDR
042	Human immunodeficiency virus (hiv) disease	10.18	<0.001	<0.001
112.0	Candidiasis of mouth	15.94	<0.001	<0.001
162.9	Malignant neoplasm of bronchus and lung unspecified	N/A	<0.001	<0.001
171.0	Malignant neoplasm of connective and other soft tissue of head face and neck	N/A	<0.001	<0.001
171.6	Malignant neoplasm of connective and other soft tissue of pelvis	N/A	<0.001	<0.001
171.9	Malignant neoplasm of connective and other soft tissue site unspecified	N/A	<0.001	<0.001
174.8	Malignant neoplasm of other specified sites of female breast	40.57	<0.001	<0.001
174.9	Malignant neoplasm of breast (female) unspecified site	20.87	<0.001	<0.001
176.0	Kaposi's sarcoma skin	N/A	<0.001	<0.001
176.1	Kaposi's sarcoma soft tissue	N/A	<0.001	<0.001
176.4	Kaposi's sarcoma lung	N/A	<0.001	<0.001
176.8	Kaposi's sarcoma other specified sites	N/A	<0.001	<0.001
179	Malignant neoplasm of uterus-part unspecified	N/A	<0.001	<0.001
182.0	Malignant neoplasm of corpus uteri except isthmus	75.35	<0.001	<0.001
183.0	Malignant neoplasm of ovary	N/A	<0.001	<0.001
197.0	Secondary malignant neoplasm of lung	63.76	<0.001	<0.001
197.6	Secondary malignant neoplasm of retroperitoneum and peritoneum	N/A	<0.001	<0.001
197.7	Malignant neoplasm of liver secondary	38.64	<0.001	<0.001
198.5	Secondary malignant neoplasm of bone and bone marrow	21.74	<0.001	<0.001
198.89	Secondary malignant neoplasm of other specified sites	92.74	<0.001	<0.001
199.1	Other malignant neoplasm of unspecified site	25.60	<0.001	<0.001
284.8	Other specified aplastic anemias	17.39	<0.001	<0.001
285.1	Acute posthemorrhagic anemia	13.25	<0.001	<0.001
285.9	Anemia unspecified	3.15	<0.001	<0.001
287.5	Thrombocytopenia unspecified	5.58	<0.001	<0.001
288.0	Agranulocytosis	52.17	<0.001	<0.001
288.9	Unspecified disease of white blood cells	N/A	<0.001	<0.001
486	Pneumonia organism unspecified	3.44	<0.001	<0.001
584.9	Acute renal failure unspecified	3.76	<0.001	<0.001
614.6	Pelvic peritoneal adhesions female (postoperative) (postinfection)	37.68	<0.001	<0.001
707.03	Chronic ulcer of skin, lower back	63.76	<0.001	<0.001
780.6	Fever and other physiologic disturbances of temperature regulation	3.08	<0.001	<0.001
799.4	Cachexia	14.49	<0.001	<0.001
136.3	Pneumocystosis	31.88	<0.001	<0.001
158.0	Malignant neoplasm of retroperitoneum	N/A	<0.001	<0.001
176.3	Kaposi's sarcoma gastrointestinal sites	N/A	<0.001	<0.001
176.5	Kaposi's sarcoma lymph nodes	N/A	<0.001	<0.001
197.2	Secondary malignant neoplasm of pleura	N/A	<0.001	<0.001
288.00	Neutropenia, unspecified	16.23	<0.001	<0.001
233.0	Carcinoma in situ of breast	21.25	<0.001	<0.001
262	Other severe protein-calorie malnutrition	21.25	<0.001	<0.001
038.9	Unspecified septicemia	7.04	<0.001	<0.001
518.81	Acute respiratory failure	5.52	<0.001	<0.001
200.18	Lymphosarcoma involving lymph nodes of multiple sites	N/A	<0.001	<0.001
789.30	Abdominal or pelvic swelling mass or lump unspecified site	13.91	<0.001	<0.001
284.89	Red cell aplasia (acquired)(adult) (with thymoma)	52.17	<0.001	<0.001
783.7	Adult failure to thrive	26.08	<0.001	<0.001
182.8	Malignant neoplasm of other specified sites of body of uterus	N/A	<0.001	<0.001
173.8	Other malignant neoplasm of other specified sites of skin	46.37	<0.001	<0.001
282.60	Sickle-cell disease unspecified	4.59	<0.001	<0.001
585.9	Chronic kidney disease, unspecified	4.06	<0.001	<0.001
276.1	Hyposmolality and/or hyponatremia	4.14	<0.001	<0.001
785.52	Septic shock	11.59	<0.001	<0.001
995.91	Systemic inflammatory response syndrome due to infectious process without organ dysfunction	7.73	<0.001	<0.001
174.4	Malignant neoplasm of upper-outer quadrant of female breast	23.19	<0.001	<0.001

Continued on next page

Supporting Table S2 – continued from previous page

ICD-9	Description	Odds ratio	P-value	FDR
996.62	"Infection and inflammatory reaction due vascular device, implant and graft"	23.19	<0.001	<0.001
682.2	Cellulitis and abscess of trunk	9.11	<0.001	<0.001
995.92	Systemic inflammatory response syndrome due to infectious process with organ dysfunction	9.11	<0.001	<0.001
159.0	Malignant neoplasm of intestinal tract part unspecified	N/A	<0.001	<0.001
171.8	Malignant neoplasm of other specified sites of connective and other soft tissue	N/A	<0.001	<0.001
174.0	Malignant neoplasm of nipple and areola of female breast	N/A	<0.001	<0.001
176.2	Kaposi's sarcoma palate	N/A	<0.001	<0.001
198.82	Secondary malignant neoplasm of genital organs	N/A	<0.001	<0.001
174.5	Malignant neoplasm of lower-outer quadrant of female breast	40.57	<0.001	<0.001
180.9	Malignant neoplasm of cervix uteri unspecified site	40.57	<0.001	<0.001
611.8	Other specified disorders of breast	40.57	<0.001	<0.001
285.22	Anemia in neoplastic disease	13.04	<0.001	<0.001
451.11	Phlebitis and thrombophlebitis of femoral vein (deep) (superficial)	13.04	<0.001	<0.001
285.29	Anemia of other chronic illness	6.96	<0.001	<0.001
518.89	Other diseases of lung not elsewhere classified	6.96	<0.001	<0.001
276.7	Hyperpotassemia	7.97	<0.001	<0.001
511.9	Unspecified pleural effusion	4.83	<0.001	<0.001
585	Chronic kidney disease (ckd)	8.28	<0.001	<0.001
785.6	Enlargement of lymph nodes	5.38	<0.001	<0.001
790.7	Bacteremia	5.38	<0.001	<0.001
191.9	Malignant neoplasm of brain unspecified site	20.29	<0.001	<0.001
276.5	Volume depletion disorder	4.77	<0.001	<0.001
031.2	Disseminated mycobacterium	34.78	<0.001	<0.001
196.3	Secondary and unspecified malignant neoplasm of lymph nodes of axilla and upper limb	34.78	<0.001	<0.001
366.16	Senile nuclear sclerosis	34.78	<0.001	<0.001
783.21	Loss of weight	5.35	<0.001	<0.001
157.9	Malignant neoplasm of pancreas part unspecified	N/A	<0.001	<0.001
159.9	Malignant neoplasm of ill-defined sites within the digestive organs and peritoneum	N/A	<0.001	<0.001
170.9	Malignant neoplasm of bone and articular cartilage site unspecified	N/A	<0.001	<0.001
171.3	Malignant neoplasm of connective and other soft tissue of lower limb including hip	N/A	<0.001	<0.001
171.4	Malignant neoplasm of connective and other soft tissue of thorax	N/A	<0.001	<0.001
197.4	Secondary malignant neoplasm of small intestine including duodenum	N/A	<0.001	<0.001
356.4	Idiopathic progressive polyneuropathy	N/A	<0.001	<0.001
576.1	Cholangitis	N/A	<0.001	<0.001
070.30	Viral hepatitis b without hepatic coma acute or unspecified without hepatitis delta	7.45	<0.001	<0.001
185	Malignant neoplasm of prostate	7.45	<0.001	<0.001
453.8	Embolism and thrombosis of other specified veins	7.45	<0.001	<0.001
518.0	Pulmonary collapse	4.06	<0.001	<0.001
998.59	Other postoperative infection	4.19	<0.001	<0.001
998.11	Hemorrhage complicating a procedure	6.52	<0.001	<0.001
112.84	Candidal esophagitis	10.14	<0.001	0.001
155.2	Malignant neoplasm of liver not specified as primary or secondary	28.98	<0.001	0.001
180.8	Malignant neoplasm of other specified sites of cervix	28.98	<0.001	0.001
199.0	Disseminated malignant neoplasm	28.98	<0.001	0.001
202.90	Other and unspecified malignant neoplasms of lymphoid and histiocytic tissue unspecified site	28.98	<0.001	0.001
158.8	Malignant neoplasm of specified parts of peritoneum	N/A	<0.001	0.002
158.9	Malignant neoplasm of peritoneum unspecified	N/A	<0.001	0.002
170.6	Malignant neoplasm of pelvic bones sacrum and coccyx	N/A	<0.001	0.002
171.5	Malignant neoplasm of connective and other soft tissue of abdomen	N/A	<0.001	0.002
180.0	Malignant neoplasm of endocervix	N/A	<0.001	0.002
184.0	Malignant neoplasm of vagina	N/A	<0.001	0.002
196.9	Secondary and unspecified malignant neoplasm of lymph nodes site unspecified	N/A	<0.001	0.002
198.0	Secondary malignant neoplasm of kidney	N/A	<0.001	0.002

Continued on next page

Supporting Table S2 – continued from previous page

ICD-9	Description	Odds ratio	P-value	FDR
198.81	Secondary malignant neoplasm of breast	N/A	<0.001	0.002
453.42	Venous embolism and thrombosis of deep vessels of distal lower extremity	N/A	<0.001	0.002
793.4	Nonspecific abnormal findings on radiological and other examination of gastrointestinal tract	N/A	<0.001	0.002
238.0	Neoplasm of uncertain behavior of bone and articular cartilage	11.59	<0.001	0.002
294.10	Dementia in conditions classified elsewhere without behavioral disturbance	11.59	<0.001	0.002
586	Renal failure unspecified	11.59	<0.001	0.002
453.41	Venous embolism and thrombosis of deep vessels of proximal lower extremity	8.11	<0.001	0.002
623.8	Other specified noninflammatory disorders of vagina	3.25	<0.001	0.003
415.19	Other pulmonary embolism and infarction	5.80	<0.001	0.003
562.10	Diverticulosis of colon (without hemorrhage)	2.61	<0.001	0.003
789.5	Ascites	6.76	0.001	0.003
220	Benign neoplasm of ovary	14.49	0.001	0.004
239.0	Neoplasm of unspecified nature of digestive system	14.49	0.001	0.004
571.5	Cirrhosis of liver without alcohol	4.14	0.001	0.005
787.91	Diarrhea	1.86	0.001	0.005
263.9	Unspecified protein-calorie malnutrition	5.15	0.001	0.005
008.45	Intestinal infection due to clostridium difficile	4.35	0.002	0.006
218.1	Intramural leiomyoma of uterus	5.80	0.002	0.007
707.15	Ulcer of other part of foot	5.80	0.002	0.007
272.4	Other and unspecified hyperlipidemia	1.91	0.002	0.007
078.5	Cytomegaloviral disease	23.19	0.002	0.007
151.9	Malignant neoplasm of stomach unspecified site	23.19	0.002	0.007
189.0	Malignant neoplasm of kidney except pelvis	23.19	0.002	0.007
263.1	Malnutrition of mild degree	6.96	0.002	0.010
403.91	Unspecified hypertensive renal disease with renal failure	4.01	0.002	0.010
366.10	Senile cataract unspecified	2.51	0.003	0.011
786.09	Respiratory abnormality other	2.38	0.004	0.011
682.6	Cellulitis and abscess of leg except foot	2.70	0.003	0.011
153.9	Malignant neoplasm of colon unspecified site	9.66	0.003	0.012
275.42	Hypercalcemia	9.66	0.003	0.012
786.3	Hemoptysis	9.66	0.003	0.012
998.12	Hematoma complicating a procedure	9.66	0.003	0.012
053.9	Herpes zoster without complication	5.07	0.003	0.012
280.0	Iron deficiency anemia secondary to blood loss (chronic)	5.07	0.003	0.012
285.21	Anemia in chronic kidney disease	5.07	0.003	0.012
788.20	Retention of urine unspecified	5.07	0.003	0.012
593.9	Unspecified disorder of kidney and ureter	3.19	0.003	0.012
172.7	Malignant melanoma of skin of lower limb including hip	N/A	0.003	0.012
172.8	Malignant melanoma of other specified sites of skin	N/A	0.003	0.012
172.9	Melanoma of skin site unspecified	N/A	0.003	0.012
174.3	Malignant neoplasm of lower-inner quadrant of female breast	N/A	0.003	0.012
183.4	Malignant neoplasm of parametrium	N/A	0.003	0.012
183.9	Malignant neoplasm of uterine adnexa unspecified site	N/A	0.003	0.012
188.9	Malignant neoplasm of bladder part unspecified	N/A	0.003	0.012
195.2	Malignant neoplasm of abdomen	N/A	0.003	0.012
196.5	Secondary and unspecified malignant neoplasm of lymph nodes of inguinal region and lower limb	N/A	0.003	0.012
196.8	Secondary and unspecified malignant neoplasm of lymph nodes of multiple sites	N/A	0.003	0.012
198.1	Secondary malignant neoplasm of other urinary organs	N/A	0.003	0.012
198.6	Secondary malignant neoplasm of ovary	N/A	0.003	0.012
198.7	Secondary malignant neoplasm of adrenal gland	N/A	0.003	0.012
200.10	Lymphosarcoma unspecified site	N/A	0.003	0.012
202.83	Other malignant lymphomas involving intra-abdominal lymph nodes	N/A	0.003	0.012
336.3	Myelopathy in other diseases classified elsewhere	N/A	0.003	0.012
357.6	Polyneuropathy due to drugs	N/A	0.003	0.012
512.8	Other spontaneous pneumothorax	N/A	0.003	0.012
587	Renal sclerosis unspecified	N/A	0.003	0.012
593.4	Other ureteric obstruction	N/A	0.003	0.012
599.89	Other specified disorders of urinary tract	N/A	0.003	0.012
281.9	Unspecified deficiency anemia	5.80	0.004	0.012
560.1	Paralytic ileus	5.80	0.004	0.012

Continued on next page

Supporting Table S2 – continued from previous page

ICD-9	Description	Odds ratio	P-value	FDR
276.51	Dehydration	1.86	0.004	0.013
427.31	Atrial fibrillation	2.78	0.005	0.013
041.11	Methicillin susceptible staphylococcus aureus	4.51	0.005	0.013
286.9	Other and unspecified coagulation defects	4.51	0.005	0.013
780.60	Fever, unspecified	3.48	0.005	0.013
799.02	Hypoxemia	3.86	0.005	0.014
423.9	Unspecified disease of pericardium	7.25	0.005	0.015
078.11	Condyloma acuminatum	11.59	0.005	0.016
614.1	Chronic salpingitis and oophoritis	11.59	0.005	0.016
427.89	Other specified cardiac dysrhythmias	2.35	0.006	0.018
428.0	Congestive heart failure unspecified	2.19	0.006	0.018
707.9	Chronic ulcer of unspecified site	3.05	0.006	0.019
611.72	Lump or mass in breast	2.09	0.007	0.020
401.9	Unspecified essential hypertension	1.25	0.007	0.020
403.90	Unspecified hypertensive renal disease without renal failure	2.77	0.007	0.020
620.2	Other and unspecified ovarian cyst	2.77	0.007	0.020
356.9	Unspecified idiopathic peripheral neuropathy	3.57	0.007	0.021
276.50	Volume depletion, unspecified	4.06	0.007	0.021
455.0	Internal hemorrhoids without complication	2.23	0.008	0.022
202.80	Other malignant lymphomas unspecified site	5.80	0.009	0.023
787.20	Dysphagia, unspecified	5.80	0.009	0.023
578.9	Hemorrhage of gastrointestinal tract unspecified	2.35	0.010	0.026
455.6	Unspecified hemorrhoids without complication	2.76	0.010	0.026
492.8	Other emphysema	3.69	0.010	0.028
218.2	Subserous leiomyoma of uterus	7.73	0.011	0.029
276.6	Fluid overload disorder	7.73	0.011	0.029
686.1	Pyogenic granuloma of skin and subcutaneous tissue	7.73	0.011	0.029
789.59	Other ascites	7.73	0.011	0.029
038.49	Other septicemia due to gram-negative organisms	17.39	0.011	0.033
041.04	Streptococcus infection in conditions classified elsewhere and of unspecified site streptococcus group d [enterococcus]	17.39	0.011	0.033
041.09	Streptococcus infection in conditions classified elsewhere and of unspecified site other streptococcus	17.39	0.011	0.033
070.1	Viral hepatitis a without hepatic coma	17.39	0.011	0.033
117.5	Cryptococcosis	17.39	0.011	0.033
426.4	Right bundle branch block	17.39	0.011	0.033
512.1	Iatrogenic pneumothorax	17.39	0.011	0.033
519.1	Other diseases of trachea and bronchus not elsewhere classified	17.39	0.011	0.033
583.9	Nephritis and nephropathy not specified as acute or chronic with unspecified pathological lesion in kidney	17.39	0.011	0.033
584.8	Acute renal failure with other specified pathological lesion in kidney	17.39	0.011	0.033
610.2	Fibroadenosis of breast	17.39	0.011	0.033
627.3	Postmenopausal atrophic vaginitis	17.39	0.011	0.033
785.51	Cardiogenic shock	17.39	0.011	0.033
789.33	Abdominal or pelvic swelling mass or lump right lower quadrant	17.39	0.011	0.033
998.51	Infected postoperative seroma	17.39	0.011	0.033
284.1	Pancytopenia	4.83	0.014	0.044
416.8	Other chronic pulmonary heart diseases	4.83	0.014	0.044
453.40	Venous embolism and thrombosis of unspecified deep vessels of lower extremity	4.83	0.014	0.044
528.9	Other and unspecified diseases of the oral soft tissues	4.83	0.014	0.044
591	Hydronephrosis	4.83	0.014	0.044
599.7	Hematuria	2.14	0.014	0.045
585.6	End stage renal disease	3.86	0.015	0.048
998.89	Other specified complications of procedures not elsewhere classified	3.86	0.015	0.048
458.9	Hypotension unspecified	2.52	0.016	0.048
782.3	Edema	2.36	0.017	0.049
