# Supplementary material for: Discovering Disease Associations by Integrating Electronic Clinical Data and Medical Literature
Source: PLoS One. 2011 Jun 23;6(6):e21132. doi: 10.1371/journal.pone.0021132 (PMC3121722; doi:10.1371/journal.pone.0021132)
Supplement: Table S3 — Significantly associated diseases with toxoplasmosis, compared to the influenza control cohort (FDR 0.05). If there are no patients with a diagnosis code in the control groups, odds ratio is not calculated (i.e. N/A). (PDF) [file pone.0021132.s006.pdf]

Supporting Table S3 . Significantly associated diseases with toxoplasmosis, compared to the influenza control cohort (FDR < 0.05). If there are no patients with a diagnosis code in the control groups, odds ratio is not calculated (i.e. N/A).

ICD-9	Description	Odds ratio	P-value	FDR
042	Human immunodeficiency virus (hiv) disease	28.25	<0.001	<0.001
112.0	Candidiasis of mouth	10.86	<0.001	<0.001
117.5	Cryptococcosis	N/A	<0.001	<0.001
130.0	Meningoencephalitis due to toxoplasmosis	N/A	<0.001	<0.001
130.2	Chorioretinitis due to toxoplasmosis	N/A	<0.001	<0.001
130.7	Toxoplasmosis of other specified sites	N/A	<0.001	<0.001
130.8	Multisystemic disseminated toxoplasmosis	N/A	<0.001	<0.001
305.60	Nondependent cocaine abuse unspecified use	8.25	<0.001	<0.001
345.90	Epilepsy unspecified without intractable epilepsy	6.24	<0.001	<0.001
584.9	Acute renal failure unspecified	3.77	<0.001	<0.001
780.39	Other convulsions	6.59	<0.001	<0.001
323.9	Unspecified cause of encephalitis	29.94	<0.001	<0.001
276.1	Hyposmolality and/or hyponatremia	5.23	<0.001	<0.001
285.29	Anemia of other chronic illness	7.48	<0.001	<0.001
136.3	Pneumocystosis	24.95	<0.001	<0.001
363.00	Focal chorioretinitis unspecified	112.26	<0.001	<0.001
348.8	Other conditions of brain	32.74	<0.001	<0.001
364.3	Unspecified iridocyclitis	21.38	<0.001	<0.001
130.3	Myocarditis due to toxoplasmosis	N/A	<0.001	<0.001
263.0	Malnutrition of moderate degree	7.48	<0.001	<0.001
078.5	Cytomegaloviral disease	10.39	<0.001	<0.001
348.5	Cerebral edema	37.42	<0.001	<0.001
294.10	Dementia in conditions classified elsewhere without behavioral disturbance	7.10	<0.001	<0.001
362.9	Unspecified retinal disorder	7.80	<0.001	<0.001
070.30	Viral hepatitis b without hepatic coma acute or unspecified without hepatitis delta	22.45	<0.001	<0.001
321.0	Cryptococcal meningitis	N/A	<0.001	<0.001
112.84	Candidal esophagitis	10.69	<0.001	<0.001
324.0	Intracranial abscess	46.78	<0.001	<0.001
305.1	Nondependent tobacco use disorder	3.14	<0.001	<0.001
008.45	Intestinal infection due to clostridium difficile	5.72	<0.001	<0.001
799.4	Cachexia	7.65	<0.001	<0.001
054.9	Herpes simplex without complication	5.56	<0.001	<0.001
707.03	Chronic ulcer of skin, lower back	8.32	<0.001	<0.001
311	Depressive disorder not elsewhere classified	2.20	<0.001	<0.001
054.19	Other genital herpes	74.84	<0.001	<0.001
322.9	Meningitis unspecified	74.84	<0.001	<0.001
070.70	Unspecified viral hepatitis c without hepatic coma	7.88	<0.001	<0.001
518.81	Acute respiratory failure	3.01	<0.001	<0.001
431	Intracerebral hemorrhage	12.47	<0.001	<0.001
011.90	Unspecified pulmonary tuberculosis confirmation unspecified	18.71	<0.001	<0.001
348.9	Unspecified condition of brain	18.71	<0.001	<0.001
031.2	Disseminated mycobacterium	37.42	<0.001	0.001
284.8	Other specified aplastic anemias	8.19	<0.001	0.001
331.4	Obstructive hydrocephalus	8.19	<0.001	0.001
239.6	Neoplasm of unspecified nature of brain	15.59	<0.001	0.002
693.0	Dermatitis due to drugs and medicines taken internally	6.24	<0.001	0.002
298.9	Unspecified psychosis	5.26	<0.001	0.002
130.1	Conjunctivitis due to toxoplasmosis	N/A	<0.001	0.002
269.9	Unspecified nutritional deficiency	N/A	<0.001	0.002
363.20	Chorioretinitis unspecified	N/A	<0.001	0.002
655.41	Suspected damage to fetus from other disease in the mother affecting management of mother with delivery	N/A	<0.001	0.002
263.8	Other protein-calorie malnutrition	13.36	<0.001	0.002
296.20	Major depressive affective disorder single episode unspecified degree	3.47	<0.001	0.002
995.92	Systemic inflammatory response syndrome due to infectious process with organ dysfunction	3.62	<0.001	0.003
345.10	Generalized convulsive epilepsy without intractable epilepsy	4.35	<0.001	0.003
362.10	Background retinopathy unspecified	18.71	<0.001	0.003
046.3	Progressive multifocal leukoencephalopathy	56.13	<0.001	0.005
784.3	Aphasia	56.13	<0.001	0.005

Continued on next page

Supporting Table S3 – continued from previous page

ICD-9	Description	Odds ratio	P-value	FDR
285.9	Anemia unspecified	2.00	<0.001	0.007
041.4	Escherichia coli (e. coli) infection in conditions classified elsewhere and of unspecified site	3.90	<0.001	0.007
648.91	Other current conditions classifiable elsewhere of mother with delivery	4.83	<0.001	0.007
305.20	Nondependent cannabis abuse unspecified use	7.02	<0.001	0.007
202.80	Other malignant lymphomas unspecified site	6.60	<0.001	0.008
570	Acute and subacute necrosis of liver	8.50	<0.001	0.009
070.32	Chronic viral hepatitis b without hepatic coma without hepatitis delta	12.47	0.001	0.011
294.9	Unspecified persistent mental disorders due to conditions classified elsewhere	28.07	0.001	0.011
585.9	Chronic kidney disease, unspecified	2.57	0.002	0.017
486	Pneumonia organism unspecified	1.68	0.002	0.017
041.04	Streptococcus infection in conditions classified elsewhere and of unspecified site streptococcus group d [enterococcus]	5.35	0.002	0.017
276.9	Electrolyte and fluid disorders not elsewhere classified	10.69	0.002	0.018
288.50	Leukocytopenia, unspecified	10.69	0.002	0.018
369.60	Blindness one eye not otherwise specified	10.69	0.002	0.018
458.0	Orthostatic hypotension	6.68	0.002	0.018
305.63	Nondependent cocaine abuse in remission	9.36	0.002	0.019
357.9	Unspecified inflammatory and toxic neuropathies	9.36	0.002	0.019
481	Pneumococcal pneumonia [streptococcus pneumoniae pneumonia]	9.36	0.002	0.019
202.81	Other malignant lymphomas involving lymph nodes of head face and neck	18.71	0.002	0.019
296.7	Bipolar i disorder, most recent episode (or current) unspecified	6.24	0.003	0.020
599.0	Urinary tract infection site not specified	1.59	0.003	0.022
780.99	Other general symptoms	4.09	0.003	0.022
070.20	Viral hepatitis b with hepatic coma acute or unspecified without hepatitis delta	N/A	0.003	0.023
130.4	Pneumonitis due to toxoplasmosis	N/A	0.003	0.023
150.0	Malignant neoplasm of cervical esophagus	N/A	0.003	0.023
176.0	Kaposi's sarcoma skin	N/A	0.003	0.023
176.4	Kaposi's sarcoma lung	N/A	0.003	0.023
244.3	Other iatrogenic hypothyroidism	N/A	0.003	0.023
320.9	Meningitis due to unspecified bacterium	N/A	0.003	0.023
333.5	Other choreas	N/A	0.003	0.023
349.82	Toxic encephalopathy	N/A	0.003	0.023
362.11	Hypertensive retinopathy	N/A	0.003	0.023
518.1	Interstitial emphysema	N/A	0.003	0.023
584.5	Acute renal failure with lesion of tubular necrosis	4.22	0.002	0.023
345.80	Other forms of epilepsy without intractable epilepsy	14.03	0.004	0.025
661.11	Secondary uterine inertia with delivery	14.03	0.004	0.025
070.54	Chronic hepatitis c without hepatic coma	3.85	0.004	0.029
263.1	Malnutrition of mild degree	4.49	0.004	0.029
785.6	Enlargement of lymph nodes	3.74	0.005	0.031
287.5	Thrombocytopenia unspecified	2.52	0.004	0.031
293.0	Delirium due to conditions classified elsewhere	7.48	0.004	0.031
288.60	Leukocytosis, unspecified	5.20	0.005	0.032
296.30	Major depressive affective disorder recurrent episode unspecified degree	2.71	0.006	0.036
348.30	Encephalopathy unspecified	4.01	0.006	0.040
041.09	Streptococcus infection in conditions classified elsewhere and of unspecified site other streptococcus	11.23	0.006	0.041
305.03	Nondependent alcohol abuse in remission	11.23	0.006	0.041
305.21	Nondependent cannabis abuse continuous use	11.23	0.006	0.041
342.90	Unspecified hemiplegia and hemiparesis affecting unspecified side	11.23	0.006	0.041
345.3	Grand mal status epileptic	11.23	0.006	0.041
054.10	Genital herpes unspecified	6.24	0.007	0.041
646.83	Other specified antepartum complications	6.24	0.007	0.041
