# Supplementary material for: Discovering Disease Associations by Integrating Electronic Clinical Data and Medical Literature
Source: PLoS One. 2011 Jun 23;6(6):e21132. doi: 10.1371/journal.pone.0021132 (PMC3121722; doi:10.1371/journal.pone.0021132)
Supplement: Table S4 — Significantly associated diseases with toxoplasmosis, compared to the PTSD control cohort (FDR 0.05). If there are no patients with a diagnosis code in the control groups, odds ratio is not calculated (i.e. N/A). (PDF) [file pone.0021132.s007.pdf]

Supporting Table S4 . Significantly associated diseases with toxoplasmosis, compared to the PTSD control cohort (FDR < 0.05). If there are no patients with a diagnosis code in the control groups, odds ratio is not calculated (i.e. N/A).

ICD-9	Description	Odds ratio	P-value	FDR
042	Human immunodeficiency virus (hiv) disease	19.32	<0.001	<0.001
078.5	Cytomegaloviral disease	92.83	<0.001	<0.001
112.0	Candidiasis of mouth	27.85	<0.001	<0.001
117.5	Cryptococcosis	74.26	<0.001	<0.001
130.0	Meningoencephalitis due to toxoplasmosis	408.43	<0.001	<0.001
130.7	Toxoplasmosis of other specified sites	N/A	<0.001	<0.001
130.8	Multisystemic disseminated toxoplasmosis	N/A	<0.001	<0.001
276.1	Hyposmolality and/or hyponatremia	6.30	<0.001	<0.001
285.29	Anemia of other chronic illness	13.00	<0.001	<0.001
294.10	Dementia in conditions classified elsewhere without behavioral disturbance	34.04	<0.001	<0.001
323.9	Unspecified cause of encephalitis	N/A	<0.001	<0.001
486	Pneumonia organism unspecified	4.57	<0.001	<0.001
518.81	Acute respiratory failure	8.40	<0.001	<0.001
584.9	Acute renal failure unspecified	4.72	<0.001	<0.001
707.03	Chronic ulcer of skin, lower back	74.26	<0.001	<0.001
780.39	Other convulsions	3.39	<0.001	<0.001
995.92	Systemic inflammatory response syndrome due to infectious process with organ dysfunction	15.91	<0.001	<0.001
130.2	Chorioretinitis due to toxoplasmosis	N/A	<0.001	<0.001
136.3	Pneumocystosis	37.13	<0.001	<0.001
263.0	Malnutrition of moderate degree	8.57	<0.001	<0.001
780.6	Fever and other physiologic disturbances of temperature regulation	3.17	<0.001	<0.001
038.9	Unspecified septicemia	7.96	<0.001	<0.001
112.84	Candidal esophagitis	18.57	<0.001	<0.001
008.45	Intestinal infection due to clostridium difficile	8.51	<0.001	<0.001
041.04	Streptococcus infection in conditions classified elsewhere and of unspecified site streptococcus group d [enterococcus]	55.70	<0.001	<0.001
348.5	Cerebral edema	55.70	<0.001	<0.001
431	Intracerebral hemorrhage	55.70	<0.001	<0.001
364.3	Unspecified iridocyclitis	14.85	<0.001	<0.001
331.4	Obstructive hydrocephalus	21.66	<0.001	<0.001
348.8	Other conditions of brain	21.66	<0.001	<0.001
130.3	Myocarditis due to toxoplasmosis	N/A	<0.001	<0.001
276.7	Hyperpotassemia	10.44	<0.001	<0.001
799.4	Cachexia	10.44	<0.001	<0.001
693.0	Dermatitis due to drugs and medicines taken internally	12.38	<0.001	<0.001
363.00	Focal chorioretinitis unspecified	27.85	<0.001	<0.001
362.9	Unspecified retinal disorder	7.14	<0.001	<0.001
345.90	Epilepsy unspecified without intractable epilepsy	3.15	<0.001	<0.001
585.9	"Chronic kidney disease, unspecified"	4.33	<0.001	<0.001
284.8	Other specified aplastic anemias	13.00	<0.001	<0.001
584.5	Acute renal failure with lesion of tubular necrosis	13.00	<0.001	<0.001
785.52	Septic shock	13.00	<0.001	<0.001
324.0	Intracranial abscess	46.41	<0.001	<0.001
348.9	Unspecified condition of brain	46.41	<0.001	<0.001
288.50	Leukocytopenia, unspecified	N/A	<0.001	<0.001
011.90	Unspecified pulmonary tuberculosis confirmation unspecified	23.21	<0.001	0.001
287.5	Thrombocytopenia unspecified	4.13	<0.001	0.001
202.80	Other malignant lymphomas unspecified site	11.14	<0.001	0.002
263.1	Malnutrition of mild degree	11.14	<0.001	0.002
031.2	Disseminated mycobacterium	37.13	<0.001	0.003
321.0	Cryptococcal meningitis	37.13	<0.001	0.003
362.10	Background retinopathy unspecified	37.13	<0.001	0.003
041.4	Escherichia coli (e. coli) infection in conditions classified elsewhere and of unspecified site	4.42	<0.001	0.004
054.9	Herpes simplex without complication	3.93	<0.001	0.004
790.7	Bacteremia	5.30	<0.001	0.005
403.90	Unspecified hypertensive renal disease without renal failure	4.04	<0.001	0.005
070.30	Viral hepatitis b without hepatic coma acute or unspecified without hepatitis delta	7.96	<0.001	0.005

Continued on next page

Supporting Table S4 – continued from previous page

ICD-9	Description	Odds ratio	P-value	FDR
239.6	Neoplasm of unspecified nature of brain	11.60	<0.001	0.006
285.9	Anemia unspecified	2.01	<0.001	0.006
288.0	Agranulocytosis	18.57	0.001	0.006
293.0	Delirium due to conditions classified elsewhere	18.57	0.001	0.006
996.62	Infection and inflammatory reaction due vascular device, implant and graft	18.57	0.001	0.006
046.3	Progressive multifocal leukoencephalopathy	N/A	<0.001	0.007
130.1	Conjunctivitis due to toxoplasmosis	N/A	<0.001	0.007
202.81	Other malignant lymphomas involving lymph nodes of head face and neck	N/A	<0.001	0.007
269.9	Unspecified nutritional deficiency	N/A	<0.001	0.007
363.20	Chorioretinitis unspecified	N/A	<0.001	0.007
574.50	Calculus of bile duct without cholecystitis without obstruction	N/A	<0.001	0.007
655.41	Suspected damage to fetus from other disease in the mother affecting management of mother with delivery	N/A	<0.001	0.007
996.2	Mechanical complication of nervous system device implant and graft	N/A	<0.001	0.007
345.10	Generalized convulsive epilepsy without intractable epilepsy	3.71	0.001	0.008
570	Acute and subacute necrosis of liver	9.28	0.001	0.010
787.20	Dysphagia, unspecified	9.28	0.001	0.010
599.0	Urinary tract infection site not specified	1.68	0.002	0.010
403.91	Unspecified hypertensive renal disease with renal failure	5.00	0.002	0.011
263.8	Other protein-calorie malnutrition	7.74	0.002	0.012
262	Other severe protein-calorie malnutrition	12.38	0.002	0.013
276.9	Electrolyte and fluid disorders not elsewhere classified	12.38	0.002	0.013
785.6	Enlargement of lymph nodes	4.64	0.003	0.016
348.30	Encephalopathy unspecified	5.57	0.003	0.016
428.0	Congestive heart failure unspecified	2.68	0.003	0.016
041.09	Streptococcus infection in conditions classified elsewhere and of unspecified site other streptococcus	27.85	0.003	0.018
075	Infectious mononucleosis	27.85	0.003	0.018
253.6	Other disorders of neurohypophysis	27.85	0.003	0.018
284.89	Red cell aplasia (acquired)(adult) (with thymoma)	27.85	0.003	0.018
364.00	Acute and subacute iridocyclitis unspecified	27.85	0.003	0.018
785.51	Cardiogenic shock	27.85	0.003	0.018
996.81	Complications of transplanted kidney	27.85	0.003	0.018
585	Chronic kidney disease (ckd)	6.63	0.004	0.025
054.19	Other genital herpes	9.28	0.004	0.027
322.9	Meningitis unspecified	9.28	0.004	0.027
423.9	Unspecified disease of pericardium	9.28	0.004	0.027
429.3	Cardiomegaly	9.28	0.004	0.027
481	Pneumococcal pneumonia [streptococcus pneumoniae pneumonia]	9.28	0.004	0.027
780.99	Other general symptoms	4.06	0.005	0.028
507.0	Pneumonitis due to inhalation of food or vomitus	3.54	0.005	0.029
276.5	Volume depletion disorder	3.82	0.006	0.032
783.21	Loss of weight	4.28	0.007	0.035
276.0	Hyperosmolality and/or hypernatremia	7.43	0.007	0.035
646.83	Other specified antepartum complications	7.43	0.007	0.035
790.93	Elevated prostate specific antigen (psa)	7.43	0.007	0.035
648.91	Other current conditions classifiable elsewhere of mother with delivery	3.23	0.007	0.037
276.2	Acidosis	3.61	0.008	0.038
511.9	Unspecified pleural effusion	3.61	0.008	0.038
276.51	Dehydration	1.95	0.011	0.042
288.60	Leukocytosis, unspecified	4.64	0.011	0.044
591	Hydronephrosis	6.19	0.011	0.044
263.9	Unspecified protein-calorie malnutrition	5.16	0.008	0.046
995.91	Systemic inflammatory response syndrome due to infectious process without organ dysfunction	5.16	0.008	0.046
038.0	Streptococcal septicemia	13.92	0.008	0.047
038.8	Other specified septicemias	13.92	0.008	0.047
342.90	Unspecified hemiplegia and hemiparesis affecting unspecified side	13.92	0.008	0.047
426.11	First degree atrioventricular block	13.92	0.008	0.047
428.20	Unspecified systolic heart failure	13.92	0.008	0.047
783.7	Adult failure to thrive	13.92	0.008	0.047

Continued on next page

ICD-9	Description	Odds ratio	P-value	FDR
031.0	Pulmonary diseases due to other mycobacteria	N/A	0.009	0.048
038.19	Other staphylococcal septicemia	N/A	0.009	0.048
041.10	Staphylococcus infection in conditions classified elsewhere and of unspecified site staphylococcus unspecified	N/A	0.009	0.048
070.20	Viral hepatitis b with hepatic coma acute or unspecified without hepatitis delta	N/A	0.009	0.048
117.3	Aspergillosis	N/A	0.009	0.048
130.4	Pneumonitis due to toxoplasmosis	N/A	0.009	0.048
139.8	Late effects of other and unspecified infectious and parasitic diseases	N/A	0.009	0.048
150.0	Malignant neoplasm of cervical esophagus	N/A	0.009	0.048
150.8	Malignant neoplasm of other specified part of esophagus	N/A	0.009	0.048
176.0	Kaposi's sarcoma skin	N/A	0.009	0.048
176.4	Kaposi's sarcoma lung	N/A	0.009	0.048
244.3	Other iatrogenic hypothyroidism	N/A	0.009	0.048
289.3	Lymphadenitis unspecified except mesenteric	N/A	0.009	0.048
290.10	Presenile dementia uncomplicated	N/A	0.009	0.048
320.9	Meningitis due to unspecified bacterium	N/A	0.009	0.048
333.5	Other choreas	N/A	0.009	0.048
349.82	Toxic encephalopathy	N/A	0.009	0.048
356.4	Idiopathic progressive polyneuropathy	N/A	0.009	0.048
362.11	Hypertensive retinopathy	N/A	0.009	0.048
453.42	Venous embolism and thrombosis of deep vessels of distal lower extremity	N/A	0.009	0.048
518.1	Interstitial emphysema	N/A	0.009	0.048
519.2	Mediastinitis	N/A	0.009	0.048
530.4	Perforation of esophagus	N/A	0.009	0.048
707.04	Chronic ulcer of skin, hip	N/A	0.009	0.048
812.40	Fracture of unspecified part of lower end of humerus closed	N/A	0.009	0.048
