# Supplementary material for: Discovering Disease Associations by Integrating Electronic Clinical Data and Medical Literature
Source: PLoS One. 2011 Jun 23;6(6):e21132. doi: 10.1371/journal.pone.0021132 (PMC3121722; doi:10.1371/journal.pone.0021132)
Supplement: Table S5 — Significantly associated diseases with Kawasaki sdisease, compared to the influenza control cohort (FDR 0.05). If there are no patients with a diagnosis code in the control groups, odds ratio is not calculated (i.e. N/A). (PDF) [file pone.0021132.s008.pdf]

Supporting Table S5 . Significantly associated diseases with Kawasaki sdisease, compared to the influenza control cohort (FDR < 0.05). If there are no patients with a diagnosis code in the control groups, odds ratio is not calculated (i.e. N/A).

ICD-9	Description	Odds ratio	P-value	FDR
372.30	Conjunctivitis unspecified	3.70	<0.001	<0.001
714.30	Chronic or unspecified polyarticular juvenile rheumatoid arthritis	52.53	<0.001	<0.001
780.6	Fever and other physiologic disturbances of temperature regulation	1.73	<0.001	<0.001
414.11	Aneurysm of coronary vessels	72.73	<0.001	<0.001
446.0	Polyarteritis nodosa	N/A	<0.001	<0.001
746.85	Coronary artery anomaly congenital	24.24	<0.001	0.001
710.0	Systemic lupus erythematosus	6.06	<0.001	0.007
034.1	Scarlet fever	10.39	<0.001	0.015
299.00	Autistic disorder current or active state	15.15	<0.001	0.017
746.1	Tricuspid atresia and stenosis congenital	24.24	<0.001	0.022
446.7	Takayasu's disease	N/A	<0.001	0.026
782.1	Rash and other nonspecific skin eruption	1.80	<0.001	0.035
