# Supplementary material for: Discovering Disease Associations by Integrating Electronic Clinical Data and Medical Literature
Source: PLoS One. 2011 Jun 23;6(6):e21132. doi: 10.1371/journal.pone.0021132 (PMC3121722; doi:10.1371/journal.pone.0021132)
Supplement: Table S6 — Significantly associated diseases with Kawasaki disease, compared to the PTSD control cohort (FDR 0.05). If there are no patients with a diagnosis code in the control groups, odds ratio is not calculated (i.e. N/A). (PDF) [file pone.0021132.s009.pdf]

Supporting Table S6 . Significantly associated diseases with Kawasaki disease, compared to the PTSD control cohort (FDR < 0.05). If there are no patients with a diagnosis code in the control groups, odds ratio is not calculated (i.e. N/A).

ICD-9	Description	Odds ratio	P-value	FDR
079.89	Other specified viral infection	15.79	<0.001	<0.001
079.99	Unspecified viral infection	3.82	<0.001	<0.001
372.30	Conjunctivitis unspecified	9.42	<0.001	<0.001
382.9	Unspecified otitis media	6.21	<0.001	<0.001
466.19	Acute bronchiolitis due to other infectious organisms	39.09	<0.001	<0.001
714.30	Chronic or unspecified polyarticular juvenile rheumatoid arthritis	78.18	<0.001	<0.001
780.6	Fever and other physiologic disturbances of temperature regulation	6.39	<0.001	<0.001
780.60	Fever, unspecified	14.43	<0.001	<0.001
782.1	Rash and other nonspecific skin eruption	4.61	<0.001	<0.001
034.0	Streptococcal sore throat	8.75	<0.001	<0.001
519.9	Unspecified disease of respiratory system	30.07	<0.001	<0.001
786.00	Respiratory abnormality unspecified	7.65	<0.001	<0.001
691.8	Other atopic dermatitis and related conditions	7.11	<0.001	<0.001
057.9	Viral exanthem unspecified	42.10	<0.001	<0.001
074.0	Herpangina	N/A	<0.001	<0.001
414.11	Aneurysm of coronary vessels	N/A	<0.001	<0.001
691.0	Diaper or napkin rash	N/A	<0.001	<0.001
746.85	Coronary artery anomaly congenital	N/A	<0.001	<0.001
034.1	Scarlet fever	36.08	<0.001	<0.001
315.39	Other developmental speech disorder	36.08	<0.001	<0.001
079.2	Coxsackie virus infection in conditions classified elsewhere and of unspecified site	N/A	<0.001	<0.001
289.3	Lymphadenitis unspecified except mesenteric	N/A	<0.001	<0.001
446.0	Polyarteritis nodosa	N/A	<0.001	<0.001
462	Acute pharyngitis	2.28	<0.001	0.003
423.9	Unspecified disease of pericardium	10.52	<0.001	0.003
464.4	Croup	30.07	<0.001	0.005
299.00	Autistic disorder current or active state	30.07	<0.001	0.005
747.0	Patent ductus arteriosus	N/A	<0.001	0.005
746.1	Tricuspid atresia and stenosis congenital	N/A	<0.001	0.005
787.03	Vomiting alone	2.38	<0.001	0.007
783.40	Unspecified lack of normal physiological development	24.06	0.002	0.020
446.5	Giant cell arteritis	24.06	0.002	0.020
746.9	Unspecified congenital anomaly of heart	24.06	0.002	0.020
078.0	Molluscum contagiosum	10.02	0.002	0.030
474.10	Hypertrophy of tonsil with adenoids	10.02	0.002	0.030
314.1	Hyperkinesis of childhood with developmental delay	10.02	0.002	0.030
786.06	Tachypnea	10.02	0.002	0.030
372.39	Other conjunctivitis	N/A	0.003	0.034
780.31	Febrile convulsions	N/A	0.003	0.034
446.7	Takayasu's disease	N/A	0.003	0.034
447.8	Other specified disorders of arteries and arterioles	N/A	0.003	0.034
315.5	Mixed development disorder	N/A	0.003	0.034
008.61	Enteritis due to rotavirus	N/A	0.003	0.034
774.6	Unspecified fetal and neonatal jaundice	N/A	0.003	0.034
745.5	Ostium secundum type atrial septal defect	N/A	0.003	0.034
466.11	Acute bronchiolitis due to respiratory syncytial virus (rsv)	N/A	0.003	0.034
277.00	Cystic fibrosis without meconium ileus	N/A	0.003	0.034
428.9	Heart failure unspecified	N/A	0.003	0.034
767.19	Other injury to scalp due to birth trauma	N/A	0.003	0.034
710.0	Systemic lupus erythematosus	3.87	0.003	0.035
112.0	Candidiasis of mouth	4.01	0.004	0.036
783.41	Failure to thrive	7.52	0.004	0.041
684	Impetigo	12.03	0.005	0.050
