# Supplementary material for: Discovering Disease Associations by Integrating Electronic Clinical Data and Medical Literature
Source: PLoS One. 2011 Jun 23;6(6):e21132. doi: 10.1371/journal.pone.0021132 (PMC3121722; doi:10.1371/journal.pone.0021132)
Supplement: Table S7 — Significantly associated diseases with Kaposi sarcoma, compared to toxoplasmosis control cohort (FDR 0.05). If there are no patients with a diagnosis code in the control groups, odds ratio is not calculated (i.e. N/A). (PDF) [file pone.0021132.s010.pdf]

**Supporting Table S7 .** Significantly associated diseases with Kaposi sarcoma, compared to toxoplasmosis control cohort (FDR < 0.05). If there are no patients with a diagnosis code in the control groups, odds ratio is not calculated (i.e. N/A).

| ICD-9 | Description | Odds ratio | P-value | FDR |
|---|---|---|---|---|
| 176.0 | Kaposi's sarcoma skin | 23.42 | <0.001 | <0.001 |
| 182.0 | Malignant neoplasm of corpus uteri except isthmus | N/A | <0.001 | <0.001 |
| 199.1 | Other malignant neoplasm of unspecified site | 11.03 | <0.001 | <0.001 |
| 174.9 | Malignant neoplasm of breast (female) unspecified site | 11.24 | <0.001 | <0.001 |
| 179 | Malignant neoplasm of uterus-part unspecified | N/A | <0.001 | <0.001 |
| 197.0 | Secondary malignant neoplasm of lung | N/A | <0.001 | <0.001 |
| 171.9 | Malignant neoplasm of connective and other soft tissue site unspecified | N/A | <0.001 | <0.001 |
| 176.1 | Kaposi's sarcoma soft tissue | N/A | <0.001 | <0.001 |
| 197.7 | Malignant neoplasm of liver secondary | N/A | <0.001 | <0.001 |
| 282.60 | Sickle-cell disease unspecified | N/A | <0.001 | <0.001 |
| 198.89 | Secondary malignant neoplasm of other specified sites | N/A | <0.001 | 0.001 |
| 183.0 | Malignant neoplasm of ovary | 11.86 | <0.001 | 0.002 |
| 174.8 | Malignant neoplasm of other specified sites of female breast | N/A | <0.001 | 0.003 |
| 176.8 | Kaposi's sarcoma other specified sites | N/A | <0.001 | 0.003 |
| 288.00 | Neutropenia, unspecified | N/A | <0.001 | 0.003 |
| 401.9 | Unspecified essential hypertension | 1.61 | <0.001 | 0.003 |
| 614.6 | Pelvic peritoneal adhesions female (postoperative) (postinfection) | N/A | 0.002 | 0.004 |
| 198.5 | Secondary malignant neoplasm of bone and bone marrow | 9.37 | 0.004 | 0.011 |
| 171.0 | Malignant neoplasm of connective and other soft tissue of head face and neck | N/A | 0.004 | 0.014 |
| 171.6 | Malignant neoplasm of connective and other soft tissue of pelvis | N/A | 0.004 | 0.014 |
| 233.0 | Carcinoma in situ of breast | N/A | 0.004 | 0.014 |
| 623.8 | Other specified noninflammatory disorders of vagina | 8.74 | 0.006 | 0.016 |
| 176.4 | Kaposi's sarcoma lung | 5.31 | 0.007 | 0.022 |
| 162.9 | Malignant neoplasm of bronchus and lung unspecified | N/A | 0.007 | 0.022 |
| 197.6 | Secondary malignant neoplasm of retroperitoneum and peritoneum | N/A | 0.007 | 0.022 |
| 366.10 | Senile cataract unspecified | 5.00 | 0.010 | 0.034 |
| 285.9 | Anemia unspecified | 1.57 | 0.013 | 0.034 |
| 158.0 | Malignant neoplasm of retroperitoneum | N/A | 0.012 | 0.035 |
| 197.2 | Secondary malignant neoplasm of pleura | N/A | 0.012 | 0.035 |
| 455.0 | Internal hemorrhoids without complication | 4.68 | 0.015 | 0.038 |
| 733.90 | Disorder of bone and cartilage unspecified | 7.49 | 0.015 | 0.041 |
| 789.30 | Abdominal or pelvic swelling mass or lump unspecified site | 7.49 | 0.015 | 0.041 |
| 414.01 | Coronary atherosclerosis of native coronary artery | 3.12 | 0.017 | 0.046 |
| 789.00 | Abdominal pain unspecified site | 1.80 | 0.016 | 0.047 |
| 496 | Chronic airway obstruction not elsewhere classified | 4.37 | 0.022 | 0.048 |
